# Dual CSF1R inhibition and CD40 activation demonstrates anti-tumor activity in a 3D macrophage- HER2^+^ breast cancer spheroid model

**DOI:** 10.3389/fbioe.2023.1159819

**Published:** 2023-06-06

**Authors:** Manuel Rodriguez-Perdigon, Laetitia Haeni, Barbara Rothen-Rutishauser, Curzio Rüegg

**Affiliations:** ^1^ Laboratory of Experimental and Translational Oncology, Department of Oncology, Microbiology and Immunology, Faculty of Science and Medicine, University of Fribourg, Fribourg, Switzerland; ^2^ Adolphe Merkle Institute, Faculty of Science and Medicine, University of Fribourg, Fribourg, Switzerland

**Keywords:** breast cancer, macrophages, spheroids, 3D model, cancer immunotherapy, CD40, CSF1R inhibitor

## Abstract

The complex interaction between tumor-associated macrophages (TAMs) and tumor cells through soluble factors provides essential cues for breast cancer progression. TAMs-targeted therapies have shown promising clinical therapeutical potential against cancer progression. The molecular mechanisms underlying the response to TAMs-targeted therapies depends on complex dynamics of immune cross-talk and its understanding is still incomplete. *In vitro* models are helpful to decipher complex responses to combined immunotherapies. In this study, we established and characterized a 3D human macrophage-ER^+^ PR^+^ HER2^+^ breast cancer model, referred to as macrophage-tumor spheroid (MTS). Macrophages integrated within the MTS had a mixed M2/M1 phenotype, abrogated the anti-proliferative effect of trastuzumab on tumor cells, and responded to IFNγ with increased M1-like polarization. The targeted treatment of MTS with a combined CSF1R kinase inhibitor and an activating anti-CD40 antibody increased M2 over M1 phenotype (CD163^+^/CD86^+^ and CD206^+^/CD86^+^ ratio) in time, abrogated G2/M cell cycle phase transition of cancer cells, promoted the secretion of TNF-α and reduced cancer cell viability. In comparison, combined treatment in a 2D macrophage-cancer cell co-culture model reduced M2 over M1 phenotype and decreased cancer cell viability. Our work shows that this MTS model is responsive to TAMs-targeted therapies, and may be used to study the response of ER^+^ PR^+^ HER2^+^ breast cancer lines to novel TAM-targeting therapies.

## 1 Introduction

Cancer growth and progression are strongly supported by monocytes recruited early on to the tumor microenvironment (TME) during tumorigenesis. Upon recruitment, monocytes differentiate into macrophages, which in turn differentiate toward a so-called tumor-associated macrophages (TAMs) phenotype facilitating metastatic cascade of cancer cells (Lin et al., 2019). A high density of TAMs generally correlates with worse clinical outcome in cancer patients (Qian and Pollard, 2010; Herrera et al., 2013; Vinogradov, Warren, and Wei, 2014; Komohara et al., 2016; Sawa-Wejksza and Kandefer-Szerszeń, 2018). Notably TAMs may account for up to 50% of the cellular tumor mass (Solinas et al., 2009; Chanmee et al., 2014), and can acquire diverse phenotypes and functionalities given their inherent plasticity. During their homeostatic adaptation, TAMs’ phenotypic and transcriptomic traits are concurrently modulated in continuous response to local cytokines, metabolites and/or interaction with cancer cells and other immune cells of the TME. Recently, attempts to establish a consensus in unifying models of TAMs diversity based on “omics” studies, suggest that one TAMs subset can perform multiple functions and, conversely, multiple subsets can perform a specific function across different cancer types (Ma, Black, and Qian, 2022). Also, diverse TAMs’ functions based on their location relative to the tumor mass have been proposed (Yang et al., 2018). Multifaced therapeutic approaches have been developed to prevent or suppress TAMs pro-tumoral functions and thereby to potentially reduce cancer cell proliferation, survival, angiogenesis and metastasis (Xiang et al., 2021). TAMs-targeted therapies can be divided globally into two categories: 1) depleting TAMs by blocking monocyte recruitment to the tumoral microenvironment and/or reducing TAMs’ survival; 2) enforcing polarization from the M2-like pro-tumoral to a M1-like anti-tumoral phenotype (Biswas, Allavena, and Mantovani, 2013). Several repolarizing strategies such as tumour necrosis factor alpha (TNF-α) receptor activation and macrophage colony-stimulating factor 1 (M-CSF1)/CSF1 receptor (CSF1R) inhibition have revealed promising therapeutical potential *in vivo* in preclinical and clinical studies (Wang et al., 2022).

The M-CSF1/CSF1R axis pathway plays a crucial role for the differentiation and survival of macrophages (Sullivan and Pixley, 2014; Achkova and Maher, 2016). M-CSF1 is secreted by tumor cells and acts as an important regulator of tumor progression to metastasis by enhancing infiltration, survival and proliferation of M2-like macrophages (Jones and Ricardo, 2013). The cluster of differentiation 40 (CD40) molecule, a TNF-α receptor family member and receptor for CD40 ligand (CD40L), has been considered and tested as additional target to modulate macrophage behavior and anti-tumor immune response. CD40 is particularly expressed in B cells, myeloid cells and monocytes/macrophages (Piechutta and Berghoff, 2019). The activation of the CD40 pathway, mediated by either CD40L expressed in T cells or agonistic monoclonal antibody (anti-CD40 mAb), result in the upregulation of T cell co-stimulatory molecules (e.g., CD86, MHC) and the release of immunostimulatory and cytotoxic cytokines (e.g., IL-12, TNF-α) (Bennett et al., 1998; Ridge, Di Rosa, and Matzinger, 1998; Vonderheide, 2020), thereby enhancing cytotoxic T-cell activity impinging on cancer cell progression (Schoenberger et al., 1998; Quezada et al., 2004).

Among the different breast cancer subtypes, HER2^+^ breast cancer had an historically worse prognosis and high risk of metastasis in comparison to HER2^−^ breast cancer. The introduction of anti-HER2 therapy with trastuzumab (Herceptin) profoundly changed its clinical management and improved its prognosis. Unfortunately, however, HER2^+^ breast cancer also develop resistance to anti-HER2 therapy (Ran et al., 2022). Moreover, and importantly for our model choice, some HER2^+^ breast cancers have been shown to express both M-CSF1 and CSF1R fostering the crosstalk between this cancer subtype and TAMs (Riaz et al., 2021). This crosstalk between cancer cells and TAMs promotes tumor formation, progression, and resistance to anti- HER2 therapy (Morandi et al., 2011; Jones and Ricardo, 2013). Hence, we adopted HER2^+^ breast cancer for our MTS as clinically relevant model for targeted therapy and resistance.

Promising biologics and/or small molecules blocking the M-CSF1/CSF1R axis (Cannarile et al., 2017) or activating the CD40 pathway, have been developed (Djureinovic, Wang, and Kluger, 2021) and tested in preclinical and clinical studies. Recently, efforts have been made to combine both strategies in *in vivo* (Wiehagen et al., 2017; Perry et al., 2018) and clinical studies (Machiels et al., 2020). However clinical evidence of therapeutic benefits has been scant so far, owning in part to the limited understanding of the effect of these drugs on the dynamics between innate immune response, cancer cell proliferation and progression.

Clinically relevant *in vitro* models are necessary to predict human responses to drugs. Complex cancer 3D models, comprising adaptive and innate immune cells, have been elegantly developed and applied to study immune-cancer cell interactions (Vitale et al., 2022). Several studies tested the efficacy of combinations of chemotherapy and/or immunotherapy in 3D *in vitro* models (Della Corte et al., 2019; Amirghasemi et al., 2021; Helleberg Madsen et al., 2022), yet there is paucity of reports assessing the effect of combinations of TAMs-targeted therapies on macrophage-cancer cell interaction using *in vitro* models.

To address this need, here we developed an *in vitro* heterotypic human macrophage-ER^+^ PR^+^ HER2^+^ breast cancer cell 3D model, referred to as macrophage-tumor spheroid (MTS), and used it to investigate the effect of dual CSF1/CSF1R inhibition and CD40 activation. We observed that macrophages tend to localize at the periphery of the MTS; MTS treatment with a combined CSF1R kinase inhibitor and an activating anti-CD40 mAb increased M2 over M1 pro-tumoral phenotype (CD163/CD86 and CD206/CD86 marker ratios), abrogated G2/M phase transition of cancer cells, promoted the production of TNF-α and reduced cancer cell viability.

In summary, we report a well-defined and responsive *in vitro* human MTS model potentially useful to assess the effects of combinations of TAMs-targeted immunotherapies in ER^+^ PR^+^ HER2^+^ breast cancer.

## 2 Materials and methods

### 2.1 Drugs and preparation

CSF1 inhibitor or BLZ-945 (A15540-50, Hölzel Diagnostika) (0.001 g, 2.5 mm) was dissolved from a stock in vehicle solution or VH (1 mL of 1:3 mixture of THF/H_2_O v/v) and sonicated in a water bath at 50°C for 15 min. This drug concentration was further diluted in VH to 25 µM stock. Anti-human CD40 therapeutic antibody (Creative Biolabs, TAB-152) was dissolved to 22.2 µM stock solution. Further desired working concentration was obtained by dilution in the appropriate cell culture medium.

### 2.2 ELISA assay

Upon MTS treatment, 600 µL volume of supernatants, corresponding to 12 spheroids per condition, were obtained after centrifugation at 1,000 × g and 2,000 × g during 10 min for measuring IL-10 and TNF-α, respectively. Cleared supernatants were diluted 1:10 in corresponding 1x standard diluent buffer and IL-10 or TNF-α levels were assessed by ELISA (ab46034, IL-10 kit and ab181421, Human TNF alpha kit, Abcam) following manufacturer’s protocol. Standard curves were generated, and analyte concentration was assessed using a five and six parametric ELISA curve for IL-10 and TNF-α, respectively. The O.D. values were measured at 450 nm on a spectrophotometer (TECAN infinite M200PRO) for IL-10 levels; and on SpectraMax M2 microplate reader (Molecular Devices Corp.) for TNF-α levels.

### 2.3 Protein quantification of MTS

Spheroids were collected and centrifuged at 400 × g for 5 min at 4°C, and the pellets were resuspended in 1x RIPA lysis buffer (9803, Cell Signaling) and 1× protease/phosphatase inhibitor cocktail (5872; Cell Signaling). After one freeze–thaw cycle, the protein concentrations were determined with Bradford method (5000001, Bio-Rad) and measured at 595 nm with SpectraMax M2 microplate reader (Molecular Devices Corp.).

### 2.4 Cancer cell cultures

BT-474 cells (ATCC number HTB-20) were cultured in complete MEM (16140071, Gibco, LifeTechnologies) supplemented with 10% fetal bovine serum (FBS) (P40-37500, PAN-Biotech) and 1% penicillin/streptomycin (10000 U mL^−1^, 15140122, ThermoFisher Scientific). Culture Medium was changed every 3–4 days, passaged at ∼80% confluency using 0.05% Trypsin-EDTA (15400-045, Gibco, LifeTechnologies). Absence of *mycoplasma* contamination from BT-474 during the experiments was confirmed by using the PCR *mycoplasma* Test Kit I/C (PromoCell).

### 2.5 Primary human monocyte isolation

Collection and use of primary human MDMs for research work was approved by the Federal Office for Public Health Switzerland (reference number: 611-1, Meldung A110635/2). Peripheral blood mononuclear cells were isolated from buffy coats provided by the Swiss Transfusion Centre (Bern, Switzerland) following a protocol described by Barosova et al. (2020). Magnetic beads (Milteny Biotec GmbH) were used to select for CD14^+^ monocytes.

### 2.6 M2-like phenotype differentiation of monocyte-derived macrophages (MDMs)

Macrophage supplemented culture medium contained Gibco RPMI 1640 supplemented with 15% FBS (P40-37500, PAN-Biotech), 1% Penicillin-Streptomycin (10,000 U mL^−1^, 15140122, ThermoFisher Scientific), 0.01% L-glutamine 1X (25030-024, ThermoFisher Scientific) and 10 ng mL^−1^ M-CSF1 (PHC9504, ThermoFisher Scientific). For preparation of M2-like differentiation medium (dM2), 20 ng mL^−1^ IL-4 (200-04, PeproTech), IL-10 (200-10, PeproTech) and IL-13 (200-13, PeproTech) were added to macrophage supplemented culture medium. Following this protocol, the validation of M2-like differentiation in macrophages has been previously published (Rodriguez-Perdigon et al., 2022).

### 2.7 Macrophages tumor cell spheroid (MTS) formation

For MDMs differentiation, monocytes were cultured at a density of 1,000 × 10^3^ cells during 3 days in 6-well tissue culture plates (3516, Corning) in 1.5 mL with macrophage supplemented culture medium. Then, pan macrophages were harvested by using Accutase **(**A6964, Sigma) and used for spheroid formation. MTS were formed under non-adhesive conditions by seeding 4 × 10^3^ macrophages and 1 × 10^3^ BT747 tumor cells (ratio 4:1) in 200 µL of culture medium per well; and grown during 2 days in ultra-low attachment U-bottom 96-well plates (174925, Nunclon TM). MEM and dM2 culture medium, 50:50 v/v, were used for the spheroid growth (dM2-MEM).

### 2.8 2D macrophage-cancer cell co-cultures

Pan macrophages and BT-474 cell co-cultures were formed by seeding 300 × 10^3^ macrophages and 75 × 10^3^ cancer cells per well (ratio 4:1, respectively) in 12-well-plate (07-201-589, Corning) and incubated with 1 mL dM2-MEM culture medium during 2 days before drug treatment. 12-well-plate were previously pretreated with poly-D-lysine (P4707-50ML, ThermoFisher) for 20 min on the incubator at 37°C, 5% CO_2_ and 1x PBS washed before seeding macrophage-cancer cell co-cultures.

### 2.9 Repolarization of 2D monocultures of M2-like macrophages by CD40 monoclonal antibody

Pan macrophages were cultured at 80 × 10^3^ cells in 12-well-plates (07-201-589, Corning) and differentiated with 1 mL dM2 per well during 3 days. 12-well-plate were previously pretreated with poly-D-lysine (P4707-50ML, ThermoFisher Scientific) for 20 min at 37°C, 5% CO_2_ and washed with 1x PBS before seeding macrophages. Then, treatment of M2-like macrophages followed during 3 days of anti-CD40 mAb treatment (5, 50 and 250 nM) within 0.5 mL volume/well of dM2 prior to flow-cytometry assays.

### 2.10 Drug treatment of MTS

MTS were treated with 1) complete dM2-MEM medium (negative control) or 2) vehicle or VH (1:3 mixture of THF/H_2_O v/v), 3) 0.2-0.5-2 µM CSF1Ri, 4) 50 nM agonistic monoclonal antibody (anti-CD40), 5) combination of both drugs for 4 or 7 days prior to the flow-cytometry or ELISA assays, 6) 10 μg mL^−1^ of trastuzumab for 1 day, and 7) 20 ng mL^−1^ of TNF-α or/and 30 ng mL^−1^ of IFNγ for 1 day prior to the flow-cytometry assays. Half of the volume per well (100 µL) was previously removed before adding double final concentration of the desired working concentration of drug.

### 2.11 Drug treatment of 2D macrophage-cancer cell co-cultures

2D cell co-cultures were treated with 1) complete dM2-MEM medium (negative control), 2) 0.2–2 µM CSF1Ri, 3) 50 nM agonistic monoclonal antibody (anti-CD40), 4) combination of both drugs for 3 days prior to the flow-cytometry assays. Half of the volume per well (500 µL) was previously removed before adding double final concentration of the desired working concentration of drug.

### 2.12 Immunophenotyping and cell viability analyses by flow cytometry

After growth or drug treatment, MTS or 2D macrophage and cancer cell co-cultures were imaged by M5000 EVOS microscope (ThermoFisher Scientific). MTS or 2D macrophage and cancer cell co-culture were collected, gently washed with cold FACS running buffer (1% BSA, Running Buffer MACSQuant^®^, 130-092-747, Miltenyi Biotec), isolated by filtering *through 40-µm* cell strainer and centrifuged (500 RCF, 5 min at 4°C). Macrophages and cancer cells were stained with the following flow cytometry-grade antibodies at the concentrations recommended by the manufacturer: anti-CD163-BV421 (clone GHI/61; 333611, Biolegend), anti-CD206-FITC (clone 15.2, 321104, Biolegend), anti-HLADR-PE (clone L243, 307606, Biolegend), anti-CD11b-PE-Cy7 (clone ICRF44; 557743 BD), anti-CD86-APC (clone IT2.2, 305411, Biolegend), anti-CD86-PE (clone BU63, 374205, Biolegend), anti-CD40-APC (clone 5C3, 334309, Biolegend), in cold running buffer containing Propidium Iodine (PI) (BMS500PI, ThermoFisher Scientific) for dead cell exclusion. Additional untreated samples were prepared for fluorescence minus one control staining using OneComp eBeads™ compensation beads (01-1111-41, Thermo Fisher Scientific) to set up the cytometer. After antibody labelling for 20 min at 4°C in the dark, cells were centrifuged (500 × g, 5 min, 4°C) and gently washed in 1x cold FACS running buffer and stored at 4°C before data acquisition. Data were acquired using MACSQuant Analyzer 10 flow cytometer (Miltenyi Biotec) and analyzed using FlowJo Software (v10.6.2, FlowJo LLC). Debris were removed based on SSC/FSC gating and doublets were removed by FSC-H vs. FSC-A single-cell gating. For immunophenotyping study, dead cells were removed by considering only PI negative cells, followed by CD11b negative cell exclusion and estimation macrophage phenotype. For PI-based cell viability study, PI negative and positive cells were considered for living and total cell population. For macrophage and cancer cell viability estimation, a CD11b negative and positive cell exclusion followed, respectively.

### 2.13 Cancer cell cycle analysis by flow cytometry

To determine the percentage of MTS cancer cells present in each cell cycle phase, we used the Click-iT^®^ EdU Flow Cytometry Assay Kits (C10419, Gibco, Invitrogen) combined with FxCycle Violet Ready Flow Reagent or FxViol (R37166, Gibco, Invitrogen) as DNA-stain dye. After drug treatment, MTS were collected, gently washed with cold FACS running buffer (1% BSA, Running Buffer MACSQuant^®^, 130-092-747, Miltenyi Biotec), and cells were isolated by filtering through 40 μm cell strainer and centrifuged (500 × g, 5 min at 4°C). Cells were labelled with EdU 3 h before staining with Zombie Yellow Fixable viability dye or ZombieYellow (Lot. B296323; 77168, Biolegend). Fixation of the cells in paraformaldehyde was followed by permeabilization using saponin during 30 min at 4°C. Staining with Click-iT^®^ reaction cocktail, anti-CD11b-PE-Cy7 (clone ICRF44; 557743 BD), as human pan macrophages marker, and FxViol was in accordance with the manufacturer’s protocol. Cells were suspended in FACS Flow (342003, BD) for acquisition on a 5-laser Cytek Aurora (full spectrum flow cytometry system) and unmixed data were analyzed using FlowJo Software (v10.6.2, FlowJo LLC). Single color controls were stained on OneComp eBeads™ compensation beads (01-1111-41, Thermo Fisher Scientific) and used for spectral unmixing. Debris were removed using the SSC/FSC plot and doublets were removed by FSC-H vs. FSC-A single-cell gating. Dead cells were removed by ZombieYellow negative cells, followed by CD11b positive cell exclusion and estimation of cancer cell cycle phases. Autofluorescence was removed by selecting it as a fluorescence tag during unmixing.

### 2.14 Cancer MTT cell viability assay

BT-474 cells were seeded in 96-well plates (CLS3595, Corning) with a density of 6 × 10^3^ cells in 200 µL per well and preincubated overnight in MEM. After 2 days of cell growth, the medium was aspirated, and cells were treated with CSF1Ri (BLZ945) at 3-fold different concentrations (10, 30, 90, 270, 810, 2.43, 7.29, 21.87, 65.61, 196.83, and 590.49 µM) in MEM medium for 48 h. Then, the medium was discarded, and cells were incubated in 100 µL of fresh medium containing 0.5 mg mL^−1^ of 3-(4,5-dimethythiazol-2-yl)-2,5-diphenyl tetrazolium bromide (MTT, M2003, Sigma-Aldrich) for 2 h. After incubation, the medium was discarded again, the MTT formazan product was solubilized with 400 µL dimethyl sulfoxide (DMSO). The absorbance was measured at 570 nm with a spectrophotometer (TECAN infinite M200PRO).

### 2.15 Imaging of MTS

MTS were formed as previously described in *MTS formation* section and imaged in brightfield by M5000 EVOS microscope at 0, 2, 6, 24 and 48 h after mixing cells. For the imaging of 3, 6 and 9 days of MTS maturation, macrophages were pre-stained with 1x MitoTracker™ Deep Red (C34565, ThermoFisher Scientific) (Fluorescent channel) during 30 min following manufacturer’s instructions, washed 3 times in PBS and mixed with cancer cells in dM2-MEM culture medium. MTS were photographed at corresponding timings with EVOS M5000 imaging system. For the confocal imaging of MTS architecture, cancer cells were pre-stained with 1x cell proliferation staining reagent (ab176736, abcam) during 30 min following manufacturer’s instructions, 3x PBS washed and mixed with macrophages (in 1:4 ratio, respectively) in dM2-MEM culture medium. At 28 h after mixing, MTS were 3 times × PBS washed and fixed with 8% para-formaldehyde (Sigma-Aldrich 158127) during 12 h. Fixed MTS were 3 times washed in PBST, blocked in PBST containing 0.5% BSA for 2 h at room temperature (RT) and finally washed 3 times in PBST. MTS were incubated in ultra-low attachment U-bottom 96-well plates (174925, Nunclon TM) with primary CD45 Recombinant Rabbit monoclonal antibody (1:100; BL-178-12C7, TF) and 10 µM Hoechst 33342 (H3570, ThermoFisher Scientific) during 14 h at 4°C. MTS were then washed three times in PBST and incubated in the secondary antibody solution (1:200, Donkey anti-Rabbit Alexa Fluor 488, Invitrogen, ThermoFisher Scientific A21206) in the dark for 6 h at RT. After incubation, MTS were washed 3 times with PBST, and embedded and mounted in Antifade Mounting Medium with DAPI (H-1200-10, Vector Laboratories). For the confocal imaging of MTS on microfluidic devices, we transfer 24 h matured MTS from U-bottom 96-well plates (174925, Nunclon TM) to provided devices and followed same staining procedure as described previously. After secondary antibody incubation, MTS on microfluidic devices were washed three times with PBST and kept at 4°C before imaging. Imaging was performed with a Leica Stellaris 8 FALCON inverted laser point scanning confocal microscope equipped with a 405 nm diode laser, a white light laser and HyD detectors. Confocal stacks were acquired with a Leica objective Plan APO 20×/0.75 NA with water immersion at voxel xyz-dimensions 0.95 mm × 0.95 mm × 1 mm. Recordings were processed in Fiji/ImageJ and transferred to Imaris 9.6.0 software for visualization, manual cell segmentation and video production.

### 2.16 Statistical analysis

Data are presented as mean ± standard deviation in replicates of *n* = 1–4 independent experiments. Peripheral-blood monocytes were obtained and mixed from up to 5 independent donors. Data experiments were analyzed by a two-way ANOVA followed by Tukey´s multiple comparison test. All statistical analyses were performed with GraphPad Prism version 9.0.2 software (La Jolla, CA, United States). Statistical significance was assessed as *p < 0.05, **p < 0.01, ***p < 0.001 and ****p < 0.0001.

## 3 Results

### 3.1 Morphological and immunophenotypical characterization of a 3D macrophage-tumor spheroids (MTS) model

**FIGURE 1:**
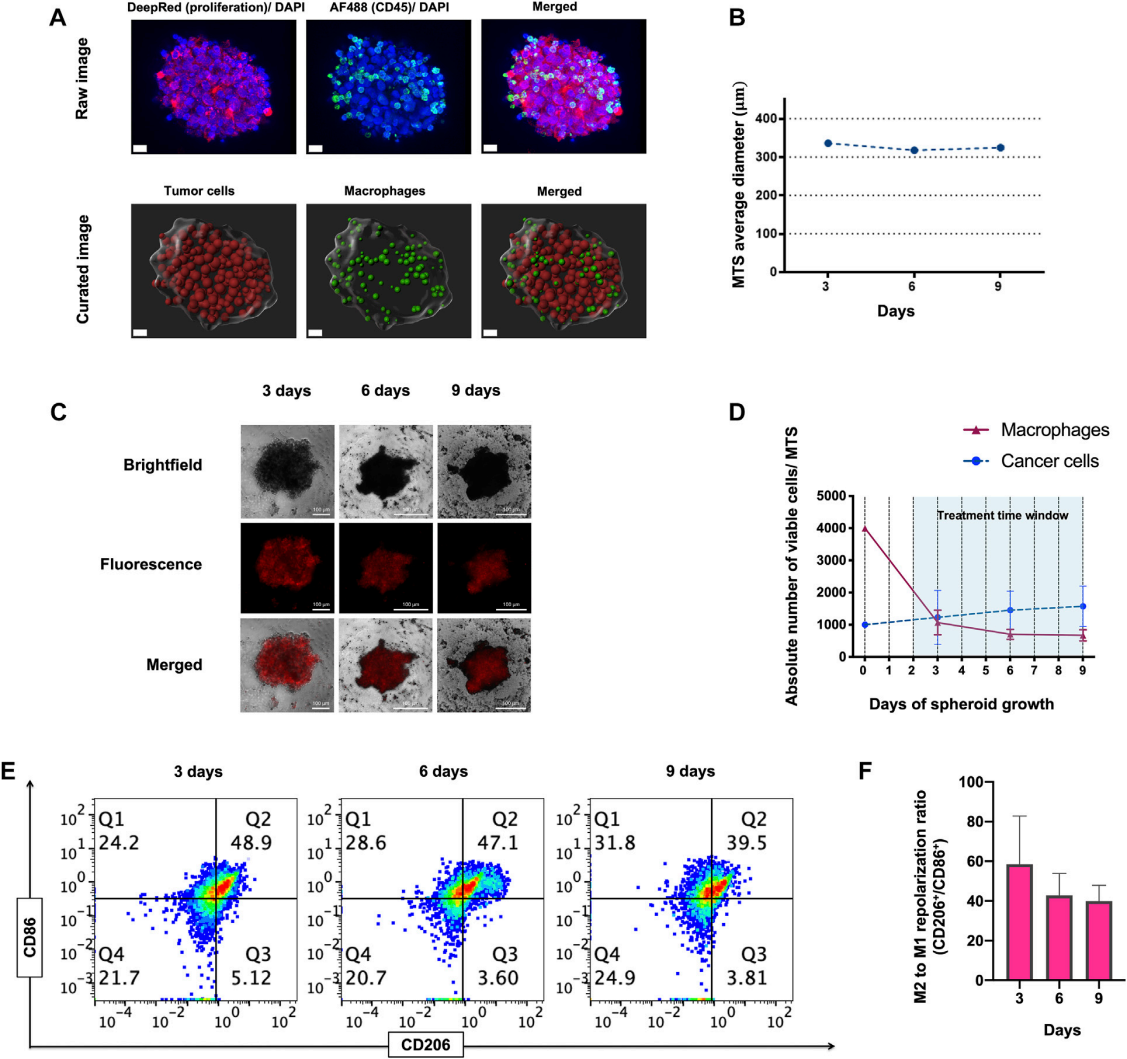
Morphology, viability and immunophenotype of macrophage-tumor spheroids (MTS) at 3, 6, and 9 days. **(A)** Confocal raw and curated images of MTS after 28 h of assembling. Nuclei are shown in blue (Hoechst), CD45^+^ macrophages in green (Alexa Fluor 488) and proliferating cancer cells in red (Deep red 633). Scale bar: 20 μm. **(B)** Average diameter of MTS over 9 days of assembling. **(C)** Representative pictures of MTS, comprising cancer cells (Brightfield channel, upper row), macrophages (Fluorescence channel, middle row), and both cells (Merged channel, lower row). A 4:1 macrophage: BT-474 cell number ratio was used. Macrophages were pre-stained with MitoTracker™ Deep Red. Scale bars: 100 μm. **(D)** Graph showing absolute numbers of viable macrophages and cancer cells per MTS. **(E)** Representative flow cytometry plots showing CD11b^+^ CD206^+^ (M2-like) and CD11b^+^ CD86^+^ (M1-like) macrophages recovered from MTS over 9 days. **(F)** Quantification of flow cytometry plots showing ratio of CD206/ CD86 expression on CD11b^+^ macrophages at 3, 6 and 9 days. Q1 represents M1-like CD86^+^ macrophages. Q2 represents mixed M2 and M1-like CD206^+^ and CD86^+^ macrophages, respectively. Q3 represents M2-like CD206^+^ macrophage. Q4 represents naïve macrophages. M2 to M1 repolarization ratio was calculated as Q1 (M2) + Q2 (M2 and M1)/Q3 (M1) + Q2 (M2 and M1) for each time point. All data are presented as the mean + standard deviation (*n* = 3).

To address our question, we engineered a 3D MTS cellular model consisting of self-aggregates of M2-like primary human macrophages and human ER^+^ PR^+^ HER2^+^ breast cancer-derived BT-474 cells. Viable MTS were produced by mixing macrophages derived by differentiation from human peripheral blood monocytes and cancer cells at a 4:1 ratio (see *materials and methods* for details). At 6 h after mixing, we observed the formation of small cellular aggregates around a core that evolved to form compact MTS within 24 h (Supplementary Figure S1A). Imaging of MTS showed that macrophages integrated into the cancer cell spheroid and tended to localize at its surface, with only rarely macrophages infiltrating the MTS core, at 28 h of assembling (Figure 1A; Supplementary Videos S1, S2). MTS displayed a rounded-aggregated morphology during 9 days of culture (Figure 1C) and a stable diameter of 325 µm in average at day 9 (Figure 1B). Macrophage viability dropped by about 75% at day 3 after assembly, in comparison to day 0 (Figure 1C). Relative (%) macrophage viability (Mean ± SD of *n* = 3: 74.36% ± 14.26%, 61.26% ± 15.03% and 63.11% ± 17.94% at 3, 6 and 9 days, respectively) as well the absolute number of viable macrophages per spheroid (Mean ± SD of *n* = 3: 1069 ± 382, 703 ± 152 and 670 ± 172 at 3, 6 and 9 days, respectively), decreased in time after (Figure 1D). This decrease in cell viability occurred mostly during the first 3 days of co-culture, and thereafter there was no further significant decrease (day 3 vs*.* day 6, *p* = 0.286; day 3 vs. day 9, *p* = 0.384) (Figure 1D). In contrast, during MTS assembly we did observe a slight increase in viable cancer cells, both in relative (%) (Mean ± SD of *n* = 3: 85.42% ± 9.07%, 91.28 ±+ 4.05% and 91.75 + 3.33% at 3, 6 and 9 days, respectively), as well as in absolute numbers (Mean ± SD of *n* = 3: 1230 ± 834, 1451 ± 589, 1575 ± 626 cells per spheroid, at 3, 6 and 9 days, respectively) (Figure 1D).

The tumor mass can contain macrophage-cancer cell ratio from 0.1:1 up to 2:1, estimated in different types of tumors and stages (van Ravenswaay Claasen et al., 1992; Lin et al., 2002; Forssell et al., 2007; Solinas et al., 2009; Chanmee et al., 2014; Knútsdóttir, Pálsson, and Edelstein-Keshet, 2014). As shown in Figure 1D, macrophage viability is reduced to one-quarter when forming MTS at day 3. This leaves a ratio of viable 1:1 macrophage-cancer cell per MTS, which is representative of a clinically relevant situation based on the previous estimations found in literature.

The BT-474 cancer cell line grows well under 2D conditions at relatively high confluency, while its growth is reduced at low cell density. When growing in 3D conditions, BT-474 spheroids retain a steady and compact size over 10 and 15 days, without evidence of cell death, consistent with a low proliferation rate under 3D conditions. As comparison, the murine cell line 4T1, a murine model for triple negative breast cancer, produced spheroids of increasing size over 10 days with a similar compacted morphology, indicative of cell growth. However, at later timepoints spheroids (i.e., 15 days), the 4T1 spheroids disaggregated, and cell death debris formed around the spheroid, [in comparison to BT-474 (Supplementary Figure S1B)].

Flow cytometry analysis showed that the M2/M1 like macrophages phenotype ratio (CD206/CD86 marker ratio) decreased along the assembling process, but this decrease was statistically non-significant (*p* = 0.187 and *p* = 0.111; day 3 vs*.* day 6 and day 9, respectively) (Figures 1E, F). Gating strategy for flow cytometry analysis for immunophenotypical characterization of MTS is shown in Supplementary Figure S1C. Overall, these results demonstrate the cellular architecture, viability and immunophenotype of a novel 3D macrophage-cancer cells *in vitro* model. We subsequently used this model for short-term drug treatments (<7 days).

### 3.2 Macrophages blunt the short-term tumor cell response to anti-HER2 therapy in the MTS model

**FIGURE 2:**
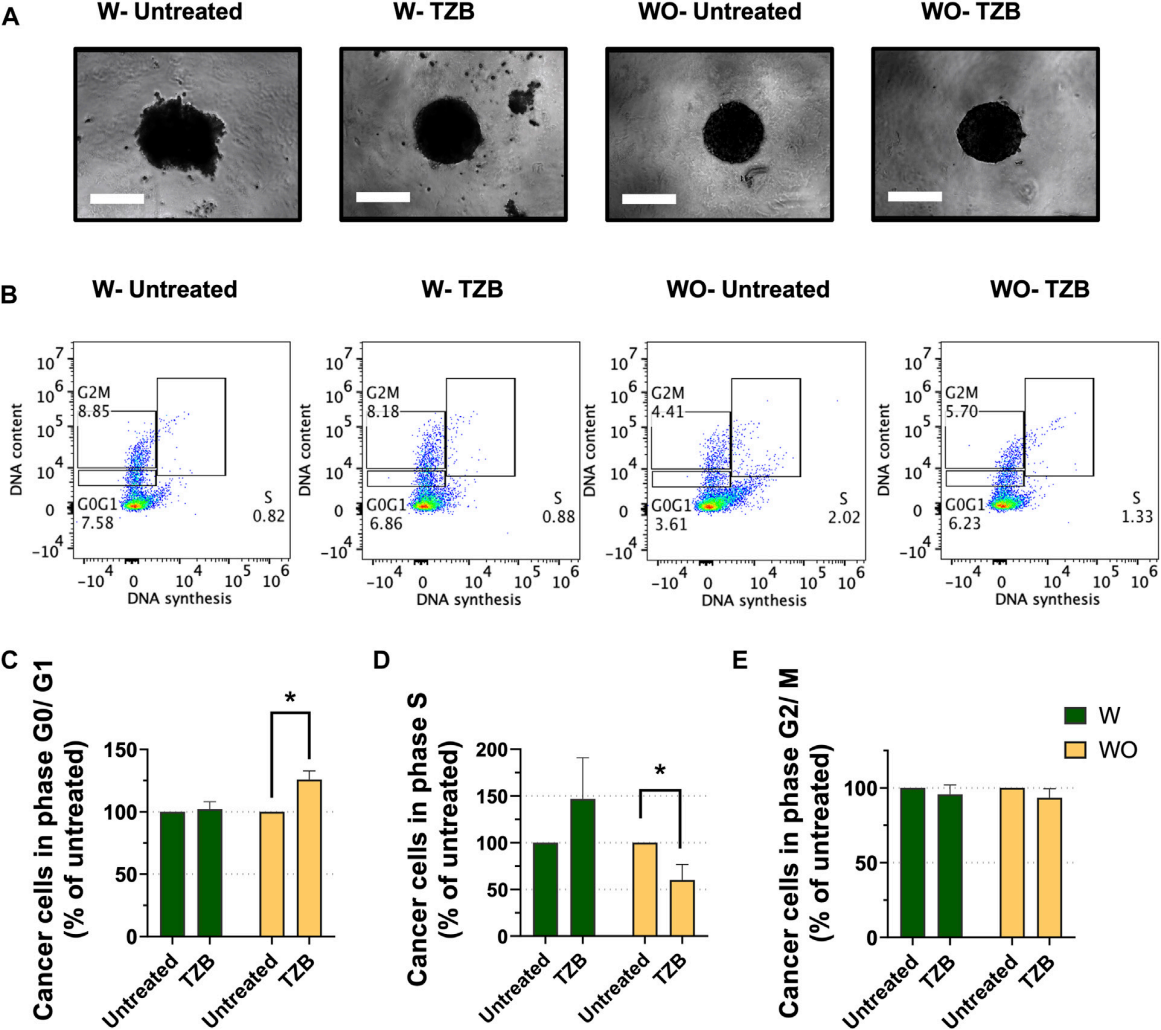
Presence of macrophages blunts the short-term response to anti-HER2 therapy in MTS model. **(A)** Representative pictures of MTS (W) and cancer cell spheroids (WO) after 7 days growth and followed by 1 day of trastuzumab treatment (TZB). Scale bar: 300 μm. **(B)** Representative flow cytometry-based cell cycle phase distribution analysis of cancer cells exposed for 1 day to TZB treatment [TZB, in the presence (W) or absence (WO) of macrophages]. **(C)** Percentage of cancer cells within G0/G1-phase, **(D)** within S-phase and **(E)** within G2/M-phase. Fractions of cancer cells in the specific cell cycle phases were normalized against untreated WO and W control. Statistical analysis was performed by two-way ANOVA followed by Tukey’s multiple comparisons. Results are considered significant with at least *p* < 0.05 (*). All data are presented as the mean + standard deviation (*n* = 2).

M2-like macrophages possess pro-tumor activities and partake on therapeutic resistance to anti-cancer therapies (Mantovani et al., 2013; Mitchem et al., 2013; Ruffell and Coussens, 2015). Trastuzumab (TZB), an antibody directed against the HER2 receptor, is commonly used to treat HER2-overexpressing (HER2^+^) breast cancer (Swain, Shastry, and Hamilton, 2022). As for other anti-cancer treatments, HER2^+^ breast cancers treated with TZB, also exhibit acquired therapeutic resistance ultimately (Chung et al., 2013; Swain et al., 2015; Wang et al., 2017). Macrophages were reported to contribute to resistance to anti-HER2 therapy (Janiszewska et al., 2021). To assess the impact of the presence of macrophages on cancer cell sensitivity to TZB in our MTS model, we co-cultured HER2^+^ BT-474 breast cancer cells with (W) and without (WO) M2-like macrophages (Figure 2A). MTS (W) and cancer cell spheroids (WO) were grown for 7 days, treated with TZB for 1 day and cells analyzed for cell cycle progression by flow cytometry.

Short-term TZB treatment of spheroids (WO) showed a significant increase of the fraction of BT-474 cells in the G0/G1-phase (*p* < 0.05), and a significant decrease of the fraction of cells in S-phase as compared to their untreated control (*p* < 0.05) (Figures 2B–D). In contrast, TZB treatment of MTS (W) did not cause significant changes in the fraction of cells in the G0/G1-or S- phases of the cell cycle compared to their untreated control (Figures 2C, D). No significant effects on G2/M-phase were observed (Figure 2E). These data indicate that macrophages blunt the short-term tumor cell response to anti-HER2 targeted therapy in MTS model, indicating that they modulate anti-tumor therapy.

### 3.3 M2 to M1-like phenotype of macrophages in MTS can be modulated by IFNγ

Cytokines limit tumour cell growth directly through anti-proliferative or pro-apoptotic activity, or indirectly by stimulating the cytotoxic anti-tumor activity of immune cells. IFNγ and TNF-α are pleiotropic cytokines with both anti- and pro-tumoral effects, depending on context and elements in the tumour microenvironment (Waters, Pober, and Bradley, 2013a; Waters, Pober, and Bradley, 2013b; Gocher, Workman, and Vignali, 2022). TNF-α can activate pathways leading to three different cellular responses: cell survival and proliferation; transcription of pro-inflammatory genes; and cell death (Waters, Pober, and Bradley, 2013b). TNF-α causes macrophage activation inducing the release of additional pro-inflammatory cytokines and enhancing their anti-tumor cytotoxic and pro-apoptotic activity (Mortara et al., 2007; Shen et al., 2018; Berraondo et al., 2019). IFNγ, produced mainly by activated T lymphocytes and natural killer cells, promotes macrophage activation, infiltration into tumor tissues and inhibition of differentiation into TAMs (Sun et al., 2014).

To explore the responsiveness of macrophages to TNF-α and IFNγ in our MTS model, we co-cultured BT-474 cells with macrophages in M2-like cell culture medium complemented with anti-inflammatory cytokines such as IL-4, IL-10 and IL-13. MTS were grown for 7 days, treated for 1 day with TNF-α and IFNγ alone or in combination. CD163/CD86 marker expression ratio (i.e., M2 to M1-like repolarization ratio) was analysed by flow cytometry. TNF-α promoted M2 to M1 repolarization, yet not significantly. IFNγ treatment alone and in combination significantly exacerbated M2 to M1 repolarization in comparison to untreated control (*p* < 0.05 and *p* < 0.01, respectively) in MTS model (Supplementary Figure S2). Altogether, from these observations, we concluded that macrophages in our MTS model are responsive to the immunomodulatory cytokines TNF-α and IFNγ.

### 3.4 Treatment of MTS with agonistic anti-CD40 mAb and CSF1Ri promotes M2-like repolarization over time

Next, we used our MTS model to investigate the effect of CSF1/CSF1R pathway inhibition and CD40 activation, alone and in combination, on macrophage M2/M1 phenotype. To determine M2 to M1-like repolarization ratio, we monitored CD206/CD86 and CD163/CD86 marker expression ratios, and to assess macrophage activation we measured HLA-DR expression by flow cytometry. At 4 days of treatment, exposure to agonistic anti-CD40 mAb and CSF1Ri, singly or in combination, did not significantly reduce the M2/M1-like polarization ratio (CD206/CD86 and CD163/CD86 marker ratios). There was, however, a trend toward reduced M2-like polarization at low CSF1Ri combined with anti-CD40 mAb, while CSF1Ri alone had no detectable effects (Supplementary Figures S3A, B). Trends were more evident at day 7, with CSF1Ri dependent effects more pronounced, while anti-CD40 mAb appeared to have little to no further effect (Supplementary Figures S3D, E). HLA-DR expression, as marker of macrophage activation, showed a trend toward increased expression in response to CSF1Ri, which was further accentuated in combination with anti-CD40 mAb treatment over 4 days (Supplementary Figure S3C). Trends were more evident at day 7 of treatment. Highest expression of HLA-DR was found in 0.5 µM CSF1Ri-anti-CD40 mAb combined treatment in comparison to untreated control, yet none of the effects were statistically significant (Supplementary Figure S3F). Vehicle only treatment (VH) did not have any significant effect on any of these parameters (Supplementary Figures S3D–F).

**FIGURE 3:**
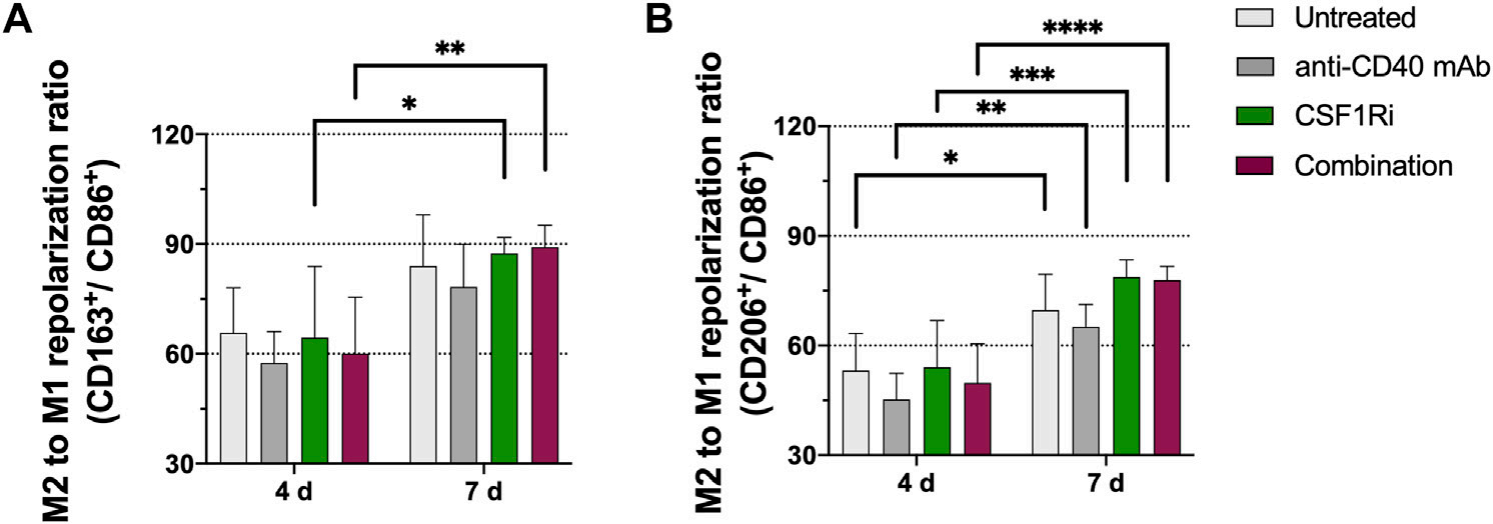
M2-like immunophenotype in MTS increases over time and is promoted by CSF1Ri and anti-CD40 mAb treatments. Representative flow cytometry plots showing ratios of **(A)** CD11b^+^ CD163^+^ (M2-like) over CD11b^+^ CD86^+^ (M1-like) macrophages; **(B)** CD11b^+^CD206^+^ (M2-like) over CD11b^+^ CD86^+^ (M1-like) macrophages from MTS at 4 and 7 days of treatment as shown: untreated, 50 nM agonistic anti-CD40 monoclonal antibody (anti-CD40 mAb), 0.5 µM inhibitor of CSF1R (CSF1Ri) and combination of both CSF1Ri and anti-CD40 mAb treatments. Statistical analysis was performed by two-way ANOVA followed by Tukey’s multiple comparisons. Results were considered significant with at least *p* < 0.05 (*), *p* < 0.01 (**), *p* < 0.001 (***) and *p* < 0.0001 (****) vs. 4 days of the homologous treatment. Results are expressed in duplicates as mean +standard deviation (*n* = 3).

When the effects of anti-CD40 mAb and 0.5 µM CSF1Ri treatments were analyzed at 4 vs. 7 days, we observed nevertheless a significant increase of the M2/M1 ratio over time. A significant increase of CD163/CD86 marker ratio (*p* < 0.05 vs. *p* < 0.01, respectively) from day 4 to day 7 occurred in the presence of single CSF1Ri and combined CSF1Ri-anti-CD40 mAb treatments (Figure 3A). Importantly, we observed a further significant increase of the CD206/CD86 marker ratio in macrophages in non-treated MTS, MTS treated with anti-CD40 mAb, CSF1Ri and a combination thereof (*p* < 0.05, *p* < 0.01; *p* < 0.001 and *p* < 0.0001; respectively) (Figure 3B).

While these experiments demonstrated that CSF1Ri and anti-CD40 mAb treatments altered polarization of MTS macrophages, the polarization effect was unexpected as macrophages responded with an increased M2-like or pro-tumoral phenotype. We then wondered whether this unexpected effect could be related to the embedding of the macrophages in 3D MTS. To address this question, we exposed macrophages co-cultured with cancer cells under 2D conditions, to CSF1Ri and anti-CD40 mAb. CSF1Ri treatment caused a progressive reduction of the M2 to M1-like repolarization in a CSF1Ri concentration dependent-manner [i.e., significant reduction of CD163/CD86 marker ratio at 2 µM CSF1Ri vs. untreated control (*p* < 0.0001, respectively)] (Supplementary Figure S4A); and significant reduction of CD206/CD86 marker ratio at 0.2 and 2 µM CSF1Ri vs. untreated control (*p* < 0.001 and *p* < 0.0001, respectively) (Supplementary Figure S4B). Addition of anti-CD40 mAb did not cause any additional effect on either M2 to M1 repolarization ratio (Supplementary Figures S4A, B). At high doses (i.e. 2 μM), CSF1Ri was cytotoxic causing approximately 20%–25% reduction in macrophage and tumor cell viability in comparison to untreated control (*p* < 0.0001, in both cases respectively) (Supplementary Figures S4C, D), also evident by the morphological degradation of the co-cultures (Supplementary Figure S4E). The experimental endpoint in cell viability studies of 2D co-cultures were chosen at 3 days when statistically significant differences were found in reduction of cancer cell viability.

As anti-CD40 mAb treatment appeared not to induce important biological effects, we tested whether macrophages responded to anti-CD40 mAb by downregulating CD40 expression. Macrophages in 2D conditions were left untreated or exposed to 5, 50 or 250 nM of anti-CD40 mAb for 3 days and subsequently analyzed by flow cytometry. Results showed that 50 nM of anti-CD40 mAb treatment downregulate CD40 expression (Supplementary Figures S5B, C), thus confirming binding and downstream activity of anti-CD40 mAb to the CD40 molecule. No major changes were observed in macrophage viability and morphology (Supplementary Figures S5A, C).

### 3.5 Combined anti-CD40 mAb and CSF1Ri treatment reduces cancer cell viability in MTS

**FIGURE 4:**
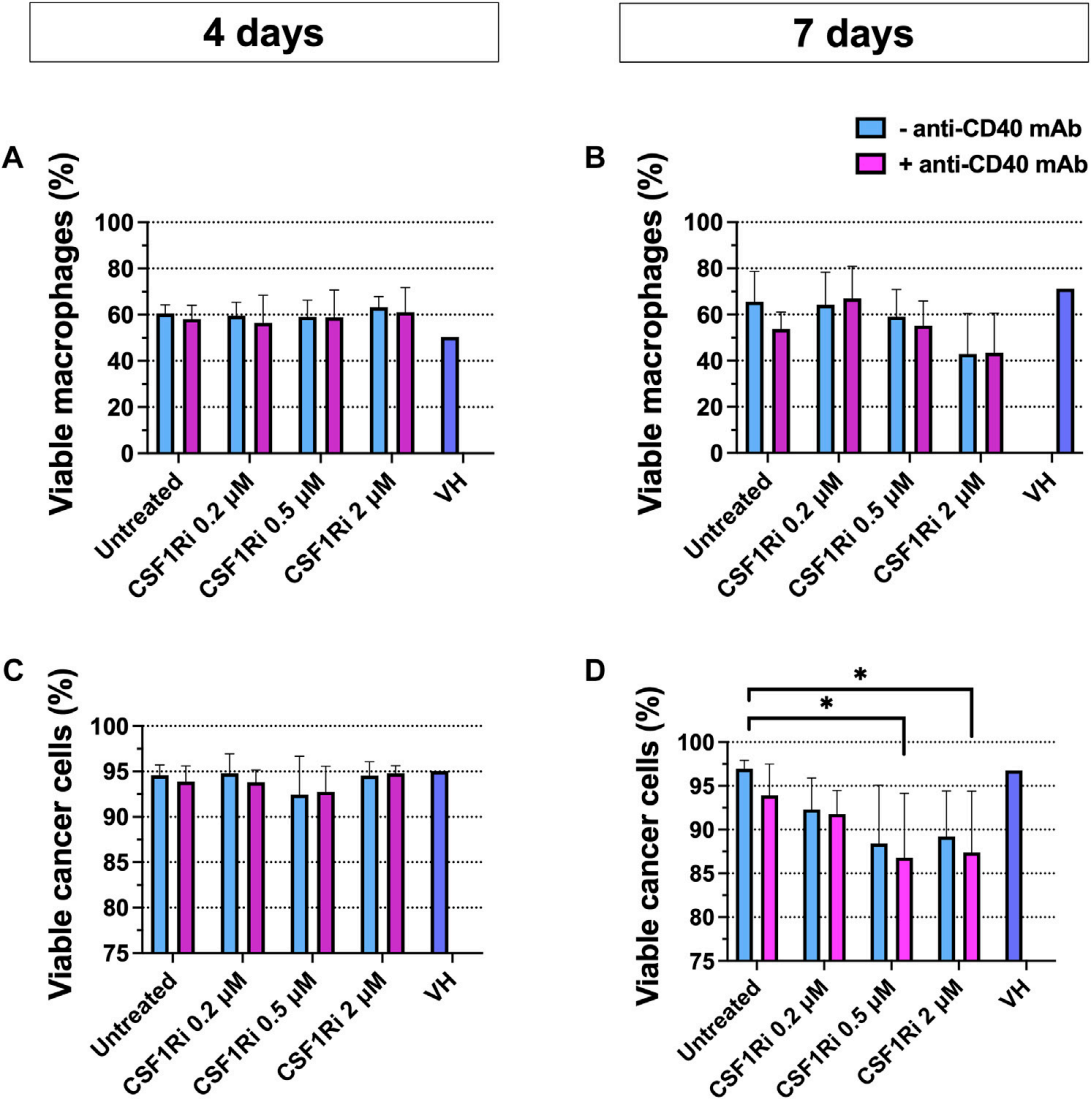
Combined anti-CD40 mAb and CSF1Ri treatment reduce cancer cell viability at 7 days treatment. Macrophage **(A,B)** and cancer cell **(C,D)** viability in MTS after 4 **(A and C)** and 7 **(B,D)** days of treatment with agonistic anti-CD40 mAb and CSF1Ri, singly and in combination, as indicated: untreated, 0.2, 0.5 and 2 µM CSF1Ri treated without (-anti-CD40 mAb) and with 50 nM (+anti-CD40 mAb). Vehicle solution or VH. Results are given in percentage viable cells) [negative Propidium Iodine (PI) stained] vs. total cancer cells; and negative PI stained- CD11b^+^ macrophage vs. total macrophage population. Statistical analysis was performed by two-way ANOVA followed by Tukey’s multiple comparisons. Results are considered significant with at least *p* < 0.05 (*) vs. untreated control. Equal volumes to 2 µM CSF1Ri solution were added as positive control of VH (1:3 mixture of THF/H_2_O v/v). Bars display data from five monocyte donors. Results are expressed in duplicates as mean + standard deviation (*n* = 3).

Next, we explored macrophage and cancer cell viability following CSF1/CSF1R pathway inhibition and CD40L/CD40 pathway activation in the MTS for 4 and 7 days. The experimental endpoint in cell viability studies of MTS were chosen at 7 days when statistically significant differences were found in cancer cell viability. Upon 4 days of treatment there was no significant drop in viability (Figures 4A, C). Upon 7 days of treatment with 0.5–2 µM CSF1Ri± anti-CD40 mAb we observed a non-significant trend of reduced macrophage viability and a significant decrease in tumor cell viability with 0.5–2 µM CSF1Ri ± anti-CD40 mAb treatment conditions (Figures 4B, D) (*p* < 0.05). Also, MTS showed a disaggregated morphology at 2 µM CSF1Ri in comparison to lower concentrations of CSF1R ± anti-CD40 mAb (Supplementary Figure S6). Vehicle solution (VH) treatment did not significantly impact the viability of either cell type (Figure 4D). No significant reduction of cancer cell viability has been reported in other 3D immune-cancer models using CSF1Ri (<0.6 µM BLZ945 concentrations) (Pyonteck et al., 2013).

As BT-474 cancer cells were reported to express CSF1R protein and mRNA (Morandi et al., 2011), albeit at lower levels compared to M2-like macrophages (Jones and Ricardo, 2013), we tested for possible direct cytotoxic effects of CSF1Ri on BT-474 cells. Indeed, we observed an inhibitory effect of CSF1Ri in 2D BT-474 cell viability (IC_50_ = 111 µM) but at doses much higher than those tested in the MTS model (≤2 µM) (Supplementary Figure S7).

These results indicate that high CSF1Ri concentrations lead to macrophage depletion only upon long-term treatments (>7 days) rather than “reprogramming” a M1/M2 phenotype in our MTS model, consistent with previously published results (Zhu et al., 2014; Hoves et al., 2018). Also, 2D macrophage treatment with *in vitro* concentrations >0.6 µM of the CSF1Ri BLZ945, resulted in TAM depletion rather in TAM reprogramming (Wei et al., 2020; Rodriguez-Perdigon et al., 2022). Our 2D and 3D results are also consistent with the fact that macrophage survival mostly depends on CSF1/CSF1R axis signaling (Chitu and Stanley, 2006), rather by activation of the CD40 signaling pathway (Rescigno et al., 1998; Djureinovic, Wang, and Kluger, 2021).

### 3.6 Combined MTS treatment with anti-CD40 mAb and CSF1Ri reduces cancer cell proliferation

**FIGURE 5:**
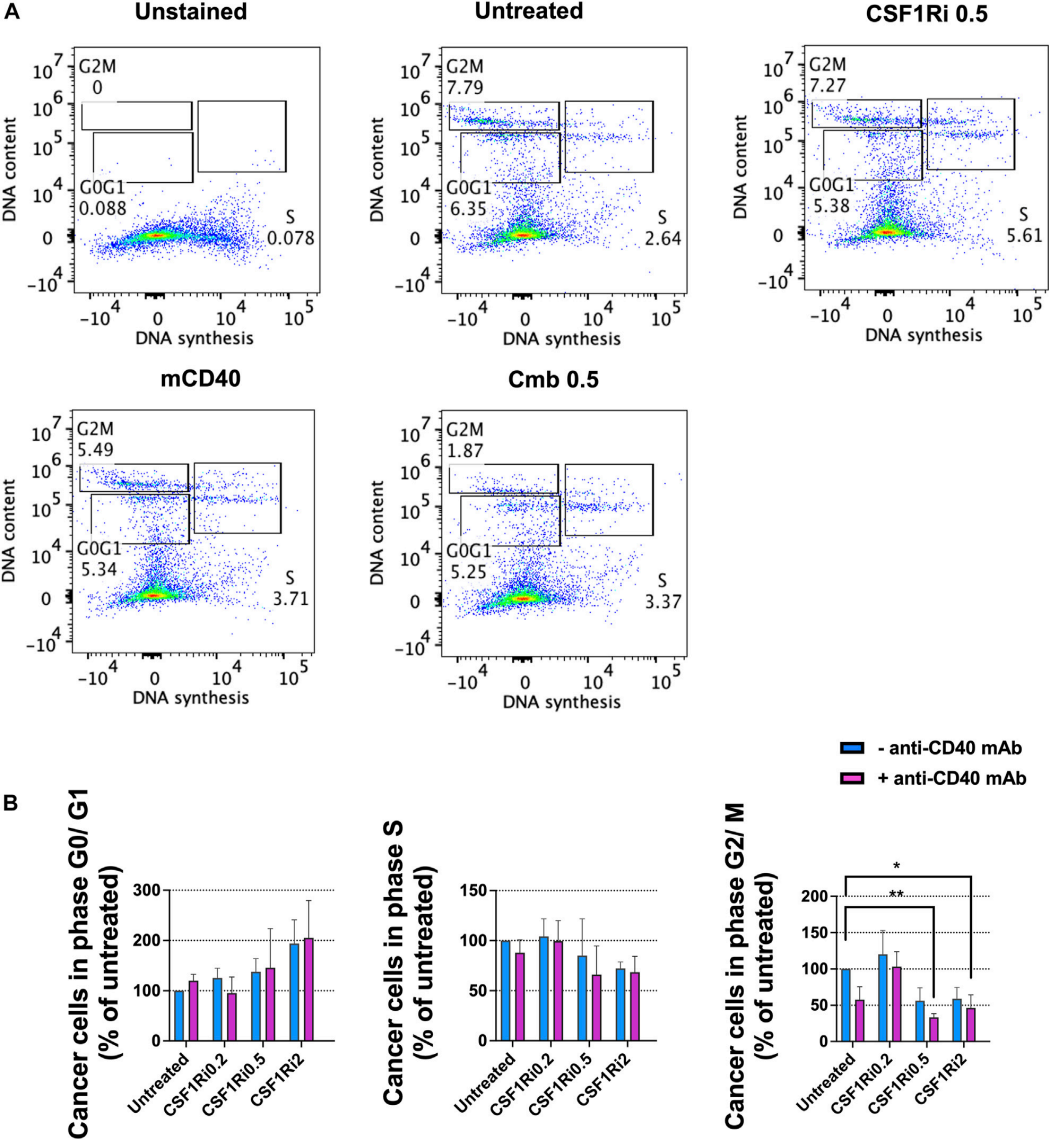
Combined treatments of MTS with anti-CD40 mAb and CSF1Ri decreases cancer cell proliferation. **(A)** Representative flow-cytometry cell cycle analysis (phase distribution) of cancer cells from MTS after 7 days of anti-CD40 mAb—CSF1Ri combined treatment: unstained, untreated, 0.5 µM CSF1R inhibitor (CSF1Ri) alone (-anti-CD40 mAb) and in combination with 50 nM of anti-CD40 mAb (+anti-CD40 mAb). **(B)** Percentage of cancer cells from MTS within G0/G1, S and G2/M phases after 7 days treated as following: untreated cultures, 0.5 µM inhibitor of CSF1R (CSF1Ri) alone (-anti-CD40 mAb) and in combination with 50 nM of anti-CD40 monoclonal antibody (+anti-CD40 mAb). The fraction of cancer cells in each cell cycle phases were normalized against the untreated controls. Statistical analysis was performed by two-way ANOVA followed by Tukey’s multiple comparisons. Results are considered significant with at least *p* < 0.05 (*) and <0.01 (**). Results are expressed in duplicates as mean +standard deviation (*n* = 4).

Next, we investigated the effect of long-term macrophage treatment with agonistic anti-CD40 mAb and CSF1Ri on cell cycle progression, as a surrogate of cell proliferation (Evan and Vousden, 2001) in the MTS model. After 7 days of treatment, we observed an increase of the fraction of cells in G0/G1-phase in either singly or combined treatment conditions vs*.* untreated control, yet non-statistically significant (Figures 5A, B). We also observed a reduction of the fraction of cells in S-phase and in either single or combo conditions also non-statistically significant (Figures 5A, B). However, combined treatment with 0.5 and 2 µM CSF1Ri and anti-CD40 mAb (50 nM) significantly reduced the percentage of cells in G2/M-phase vs*.* untreated control (*p* < 0.01 and *p* < 0.05, respectively) (Figure 5B).

From these observations, we concluded that long-term combined treatment of MTS diminished both cancer cell viability (Figure 4D) and cancer cell proliferation at > 0.5 µM of CSF1Ri - anti-CD40 mAb (Figure 5B). Upon these treatment conditions, the macrophage viability was reduced but not significantly (Figure 4B).

### 3.7 Combined treatment with anti-CD40 mAb and CSF1Ri induces secretion of TNF-α

**FIGURE 6:**
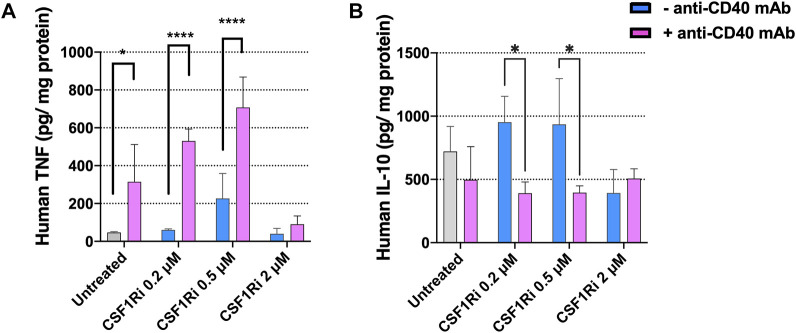
Treatment of MTS with CSF1Ri and anti-CD40 mAb increases secretion of TNF-α and reduces secretion of IL-10. **(A)** Secreted anti-tumoral cytokine TNF-α is augmented in MTS and **(B)** secreted pro-inflammatory cytokine IL-10 is reduced in MTS upon synergistic drug combination: untreated, 0.2, 0.5 and 2 µM inhibitor of CSF1R (CSF1Ri) and without (-anti-CD40 mAb) and with combination of 50 agonistic nM CD40 monoclonal antibody (+anti-CD40 mAb). Statistical analysis was performed by two-way ANOVA followed by Tukey’s multiple comparisons. Results are considered significant when *p* < 0.05 (*) and <0.0001 (****) vs. corresponding CSF1Ri treated or untreated control. Results are expressed in duplicates as mean + standard deviation (*n* = 2).

To test the effect CSF1Ri and agonistic anti-CD40 mAb treatment of MTS on the secretion of inflammatory/anti-inflammatory cytokines, we measured TNF-α and IL-10 proteins in the co-culture conditioned medium at 7 days, a timepoint where we observed a major reduction in cancer cell proliferation and viability (Figures 4D, 5B). Anti-CD40 mAb treatment caused a dose dependent induction of TNF-α secretion, and this effect was enhanced by concomitant CSF1Ri treatment (Mean ± standard deviation of *n* = 2: 46.8 ± 4.1; 314.5 ± 197.1; 226.7 ± 131.7; 707.8 ± 160.3 TNF-α pg/mg protein; untreated, anti-CD40 mAb, 0.5 µM CSF1Ri and 0.5 µM CSF1Ri -anti-CD40 mAb treatment, respectively) (Figure 6A). Upon CSF1Ri treatment there was a trend toward increased IL-10 secretion at 0.2 and 0.5 µM, though non-significantly, which was significantly blunted by anti-CD40 mAb treatment (*p* < 0.05) (Figure 6B). However, a non-significant reduction of IL-10 release was found in anti-CD40 mAb treatment alone. Previous studies in *in vitro* 3D macrophage-fibroblast-pancreatic tumor models (Helleberg Madsen et al., 2022) showed that anti-CD40 mAb activated macrophages significantly and increased the secretion of various cytokines (i.e., CCL22, IL-10 and VEGF). No 3D *in vitro* assessment of CD40 activation and CSFR1 blockade efficacy has been previously reported.

Together these results indicate that combined treatments with agonistic anti-CD40 mAb and CSF1Ri induces a pro-inflammatory immune phenotype of MTS (increased secretion of TNF-α and decreased secretion of IL-10 at 0.5 µM CSF1Ri concentration). This is consistent with previously published *in vivo* studies reporting that anti-CD40 mAb—CSF1Ri treatment significantly increase TNF-α secretion (Elgueta et al., 2009; Perry et al., 2018). *In vitro* studies showed that CD40-activated macrophages released high amount of pro-inflammatory cytokines (i.e., TNF-α) (Quezada et al., 2004); however no effect on TNF-α release was observed in CSF1Ri treated 3D tumors (Helleberg Madsen et al., 2022). High concentrations of CSF1Ri (2 µM), regardless of the presence of anti-CD40 mAb, had no effect on TNF-α or IL-10 release in comparison to untreated MTS (Figure 6). These data support the notion that sustained treatment with high CSF1Ri concentrations lead to macrophage depletion (Figure 4B; Supplementary Figure S4C), resulting in reduced cytokine production in MTS, consistent with previous reports (Zhu et al., 2014; Quail et al., 2016; Hoves et al., 2018).

## 4 Discussion

In the last decade, anti-cancer check-point inhibitors-based immunotherapy has emerged as a new standard in the treatment of numerous cancer types, most notably melanoma, head and neck, gastric, lung, renal and bladder cancers. However, not all cancer types, and not all patients within a cancer type respond to immunotherapy (Hargadon, Johnson, and Williams, 2018). In order to maximize response to immunotherapies there is increasing interest in developing combination treatments modulating the cross-communication between immune cells and tumor cells to create more favorable conditions for immunogenic response (Kirchhammer et al., 2022). 3D co-culture systems (i.e., tumor derived spheroids, heterotypic cancer spheroids, patient-derived organoids) are emerging valuable tools to decipher the dynamics between immune system and tumor progression and predict human responses to cancer immunotherapies. 3D co-culture models comprising innate immune cells, adaptive immune cells and cancer cells have been established to test tumor associated macrophages (TAMs)-targeted therapies. However, these studies mostly focused on single drug treatments and neglected the analysis of combined TAMs-targeted therapies using clinically applicable drugs, in particular CSF1R inhibitors and anti-CD40 agonists (Alvey et al., 2017; Bauleth-Ramos et al., 2020; Lee et al., 2020; Rose et al., 2020; Helleberg Madsen et al., 2022).

To address this question, we established a simple, well-defined 3D macrophage-tumor spheroid (MTS) co-culture model consisting of human monocyte-derived macrophages (MDMs) and the human ER^+^ PR^+^ HER2^+^ breast cancer line BT-474 and used it to characterize the effects of CSF1R inhibition and CD40 activation. MTS formed rapidly (1 day) (Supplementary Figure S1A) and were stable for at least 9 days (Figures 1B–D). During the initial stages of MTS assembling (between day 0 and 3), we observed a drastic decrease of macrophage viability (Figure 1D). This is likely due the death of naïve macrophages before they could establish adhesive interactions with tumor cells (Ammon et al., 2000; Schittenhelm, Hilkens, and Morrison, 2017). Surviving macrophages integrated into the cancer cell spheroid and preferentially located at its surface, while only rare macrophages infiltrated the core (Figure 1A; Supplementary Videos S1, S2), reminiscent of initial stages of tumor development (Burugu, Asleh-Aburaya, and Nielsen, 2017; Liu et al., 2021). Our MTS model may resemble tumour nodules (<500 μm diameter) comprising stromal macrophages around incipient tumour as proposed previously in other models (Mellor, Ferguson, and Callaghan, 2005; Kempf et al., 2013). Broad evidence supports the notion that location of stromal macrophages in and around the tumor nest is relevant, as it dictates the polarization and consequently the pro- or anti-tumoral function of TAMs (Rivera, and Bergers, 2013; Lendeckel et al., 2022). Some studies have reported that HER2 status was positively correlated with stromal TAMs (Gwak et al., 2015), and their relevance as prognostic marker for breast cancer patients (Medrek et al., 2012).

Although peripheral blood-derived monocytes were exposed to culture medium promoting M2-like differentiation, macrophages in MTS showed a mixed M2/M1 phenotype (Figure 1F). Importantly, upon integration they appeared to be functional as they did blunt the anti-proliferative effect of trastuzumab on BT-474 cancer cells (Figure 2) and responded to IFNγ treatment with differentiation toward M1-like phenotype (Supplementary Figure S2). MTS also responded to TAMs-targeting agonistic anti-CD40 mAb and CSF1Ri treatments, though in a more complex manner than originally anticipated. Single drug and combined treatments increased the M2 to M1-like phenotype of macrophages unexpectedly over time, in addition to a spontaneous M2 to M1-like polarization (Figure 3). Remarkably, long-term combined TAMs-targeted treatment did not cause a decrease in macrophage viability. In contrast, cancer cells from MTS exposed to combined anti-CD40 mAb and CSF1Ri treatments showed a significant reduction in viability and G2/M cell cycle progression (Figures 4D, 5B). This indirect macrophage-tumor cells cross-talk in treated MTS was paralleled by the secretion of the pro-inflammatory and cytotoxic cytokines (i.e., TNF-α) (Figure 6A). Strikingly, in 2D conditions similar treatment conditions elicited a decreased of the M2 to M1-like phenotype polarization, which was accompanied with both macrophage and cancer viability reduction (Supplementary Figure S4).

Results obtained in this study suggest the following conclusions:

i) MDMs embedded in MTS consist of a mixture of M2-like and M1-like macrophages, which, after an initial non-significant drop during MTS assembly, over time spontaneously differentiate towards more M1-like phenotype (based on CD206/CD86 marker expression) in unchanged conditioned medium. This observation is consistent with reports showing that exposure of macrophages to triple negative breast cancer cell lines but not ER^+^ PR^+^ breast cancer cell lines, skews macrophages to a M2-like phenotype (Hollmén et al., 2015; Sousa et al., 2015). Directly assigning pro- and antitumoral functions of TAMs based on the M1/M2 phenotype should be addressed with caution as macrophages can assume a more complex spectrum of phenotypes and functions depending on the context (Murray et al., 2014; A; Mantovani, 2016; Martinez and Gordon, 2014). The intrinsic and plastic modulation of the macrophage M2/M1 phenotypes by different tumor-cell lines in 3D models (Kuen et al., 2017; Raghavan et al., 2019; Madsen et al., 2021; Helleberg Madsen et al., 2022), together with factors driving the transient or stable state of macrophage activation (M2-like to M1-like phenotype) along time, call for caution when using MTS for screening drugs modulating M2/M1 phenotype. A wide range of preselected M2/M1 subsets markers should be monitored throughout the drug screening to assess the functional phenotype of TAM subsets for a given model (i.e., MTS) at any given time, in order to optimally interpret the results.
ii) MDMs embedded in MTS show plasticity: the M2/M1 phenotype can be actively modulated by IFNγ stimulation, and anti-CD40 mAb and CSF1Ri treatment. While IFNγ stimulation twisted differentiation toward M1-like phenotype, consistent with its physiological role in activating and generating cytotoxic macrophages (Castro et al., 2018), anti-CD40 mAb and CSF1Ri treatment caused increased polarization toward M2-like, rather than M1-like phenotype, which was unexpected based on *in vivo* studies (Wiehagen et al., 2017). This effect is not totally paradox as unrecognized diversity of TAMs subsets in human tumors has recently emerged (Guerriero, 2018). For example, inconsistencies of patterns in M1/M2 macrophage phenotypes in non-treated human tumors (Chevrier et al., 2017; Lavin et al., 2017; Zhang et al., 2022) in human tumors from chemo and immunotherapy responders (Bi et al., 2021; Hu et al.; A; Mantovani et al., 2017) and similar 3D macrophage-cancer cell *in vitro* models (Madsen et al., 2021). Single-cell mapping of the breast tumor microenvironment revealed the expression of pro-tumor M2-and anti-tumor M1-associated genes often occurred within the same cells, demonstrating that M1 and M2 states are not mutually exclusive as originally proposed (Azizi et al., 2018).
Strikingly, when exposed to CSF1Ri and anti-CD40 mAb macrophages co-cultured with cancer cells under 2D conditions, showed a progressive increase of the M1-like phenotype. The reasons of this 3D vs. 2D discrepancy remains unclear at this point, but similar contrasting results between 2D and 3D settings and inconsistency on M2/M1 phenotype binary classification in response to CSF1R inhibition and/or CD40 activation or other anti-cancer drugs have been reported using different cancer cell lines. For example, these unexpected phenotypic effects of M1 polarizing compounds on TAMs are suggested to reflect past rather current signalling, and timing of drug administration should be taken into consideration when evaluating novel therapies (Helleberg Madsen et al., 2022). Hetero-spheroids, comprising ovarian cancer and macrophages, treated with WNT inhibitor showed no reduction in M2-like phenotype marker. 3D differentiation and activation was similar in terms of gene expression to conventionally activated macrophages in 2D culture systems (Raghavan et al., 2019). Fibroblast-pancreatic tumor spheroids induced a M2-like phenotype on the monocytes after co-culture them (Kuen et al., 2017).
A comparison/integration of the 2D/3D results may be needed to better understand the immuno-phenotype response to TAMs targeted therapies and its anti-cancer efficacy for a given *in vitro* model. Our observation on M1/M2 phenotype vs. efficacy of CSF1Ri and CD40 ligand combination treatments, raises question on the historical TAM phenotype-function relationship and call for caution on how to monitor these treatments.
iii) MDMs embedded in MTS are functional: they blunted the short term anti-proliferative effect of trastuzumab on BT-474 cells, they produced TNF-α in response to anti-CD40 mAb and CSF1Ri treatment and they impinged on BT-747 cell viability and cell cycle progression. These effects are aligned with expected results, and particularly, they demonstrate that despite the paradox effect of CSF1Ri and anti-CD40 mAb treatment in the immunophenotype, treated macrophages acquire anti-tumor activities.
Taken together, we describe and functionally verify MTS model as useful preclinical tool for the evaluation of combined TAMs-targeted therapies, that may be used to evaluate effects of novel TAM-targeting agents on breast cancer cell behavior and fate. In view of the presented results, however, close monitoring and care should be taken into consideration to interpret results in the specific context of the immune-tumor model and avoiding extrapolating conclusions to other systems that may behave differently.

## Data Availability

The datasets presented in this study can be found in online repositories. The names of the repository/repositories and accession number(s) can be found below: https://doi.org/10.5281/zenodo.7607772.
