# Supplementary material for: Dual CSF1R inhibition and CD40 activation demonstrates anti-tumor activity in a 3D macrophage- HER2+ breast cancer spheroid model
Source: Front Bioeng Biotechnol. 2023 Jun 6;11:1159819. doi: 10.3389/fbioe.2023.1159819 (PMC10281737; doi:10.3389/fbioe.2023.1159819)
Supplement: Supplementary file 3 [file DataSheet1.docx]

Supplementary Material

Dual CSF1R inhibition and CD40 activation demonstrates anti-tumor activity in a 3D macrophage- HER2^+^ breast cancer spheroid model

Manuel Rodriguez-Perdigon^1*^, Laetitia Haeni^2^, Barbara Rothen-Rutishauser^2^ and Curzio Rüegg^1^

^1^Laboratory of Experimental and Translational Oncology, Department of Oncology, Microbiology and Immunology, Faculty of Science and Medicine, University of Fribourg, Fribourg, Switzerland

^2^Adolphe Merkle Institute, Faculty of Science and Medicine, University of Fribourg, Fribourg, Switzerland

*** Correspondence:** M. Rodriguez-Perdigon,

[mrodriguezperdigon@hotmail.com](mailto:mrodriguezperdigon@hotmail.com)

# Supplementary Data

#
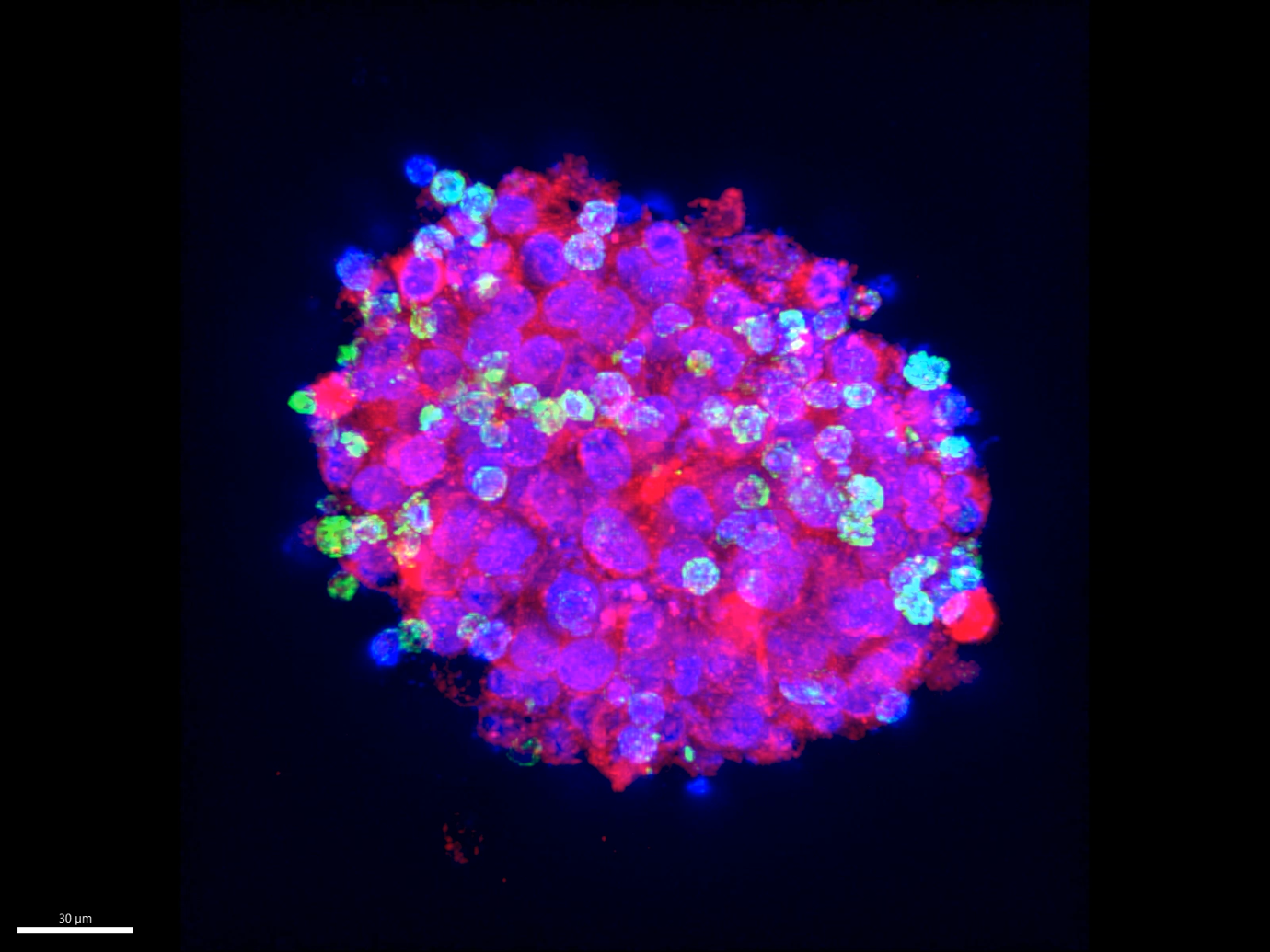


**Supplementary Video 1. Visual rotation of the macrophage- tumor spheroid (MTS).** Confocal images were used to reconstitute 100 μm thickness of MTS. The raw video shows first a 3D rotation of the MTS fragment along the Z-axis and then the top-to-bottom Z-stack migration along the Y axis. Nuclei are shown in blue (Hoechst), CD45^+^ macrophages in green (Alexa Fluor 488) and proliferating cancer cells in red (Deep red 633). Macrophages are visible mostly at the surface and absent from the inner mass of the MTS. Scale bar: 30 μm.


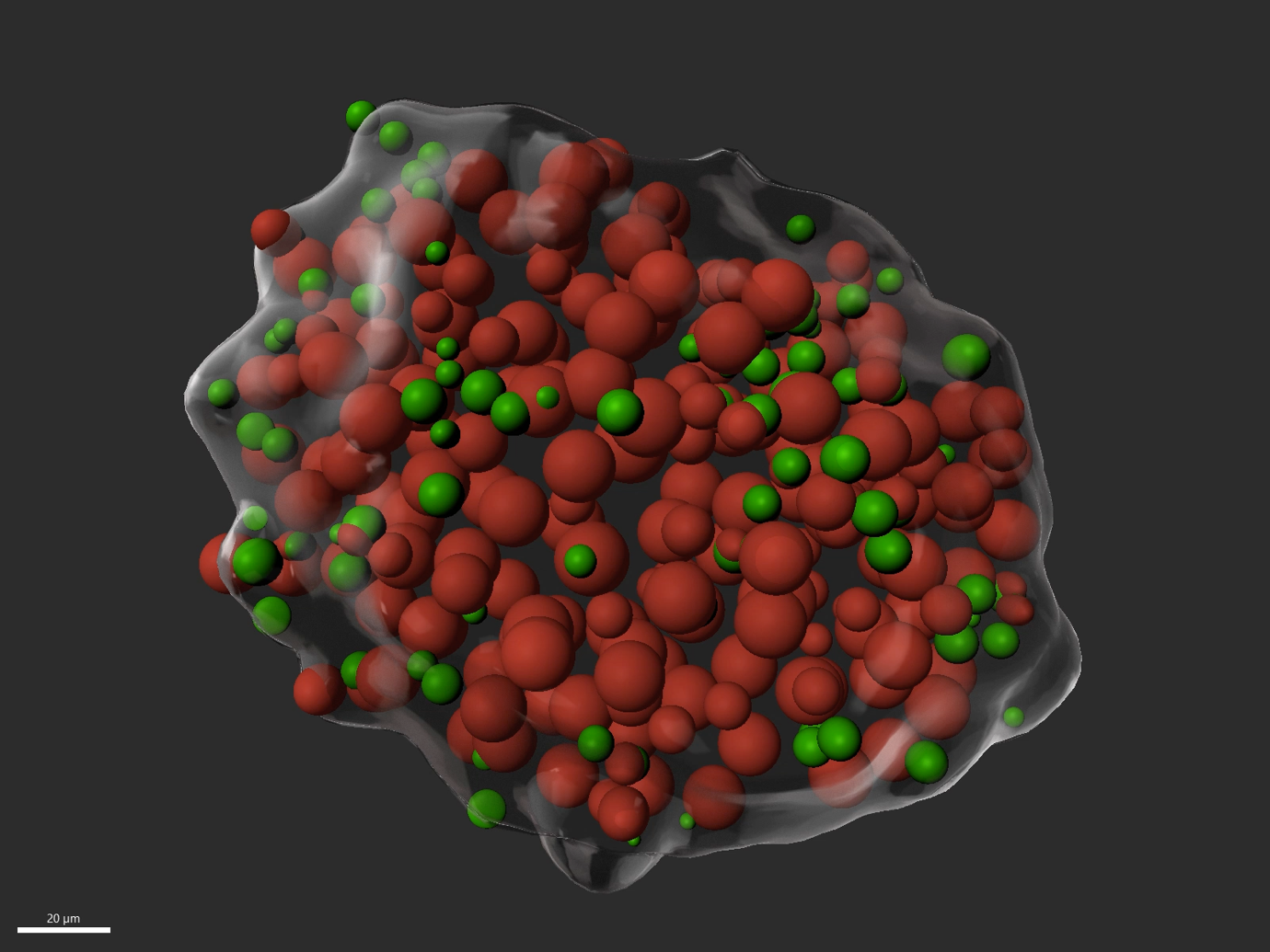


# Supplementary Video 2. Visual rotation of annotated cancer cells and macrophages of the macrophage- tumor spheroid (MTS). Confocal images were manually annotated for cancer cells (red spheres) and macrophages (green spheres) and digitally reconstituted in a 3D with MTS surface rendering (grey). Macrophages are shown first together with cancer cells (green and red spheres, respectively) and then alone (green spheres). Macrophages are present mostly at the surface of the MTS. Scale bar: 20 μm.

# Supplementary Figures

#
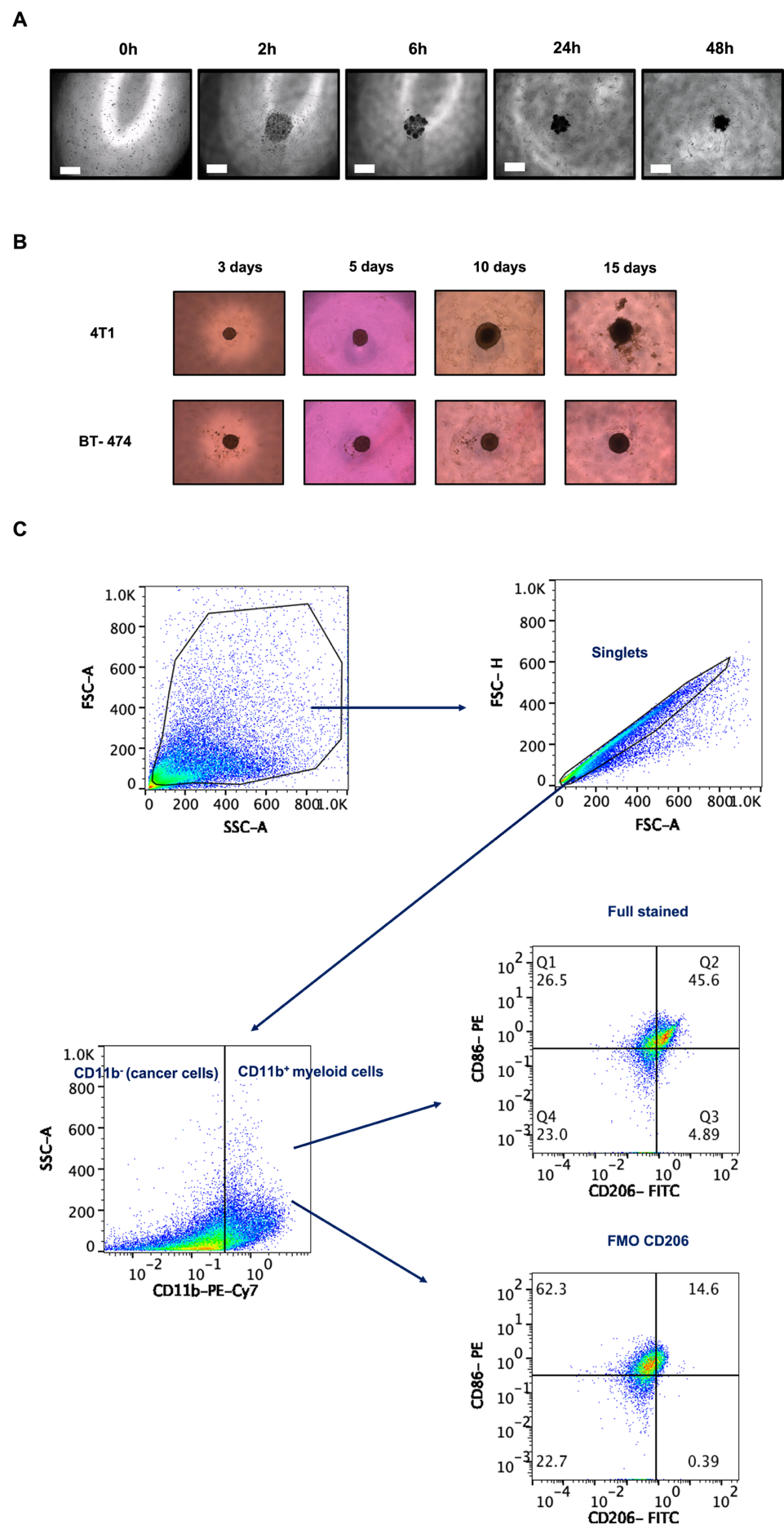


**Supplementary Figure 1. (A)** Representative images of MTS assembling process over 48h. Scale bar: 300 μm. **(B)** Morphology of breast cancer cell lines grown in 3D conditions at 3, 5, 10 and 15 days. From top to bottom: representative pictures of “spheroids” of BT474 (HER2^+^, ER^+^, PR^+^) and 4T1 (murine Triple Negative breast cancer). When growing 3D BT-474 spheroids, a relatively steady size of spheroid over 10 days is observed, and not clear sign of cell death outside the spheroid (ie. cell debris). Conversely, its counterpart 4T1 cell line, a murine triple negative breast cancer model, exhibited similar “compacted morphology” to BT-474 cell line over 10 days, but disaggregated (ie. cell dead debris around spheroid mass) at day 15. Spheroids were formed under non-adhesive conditions by seeding 1.2×10E2 cells in 200 μL of culture medium per well in ultra-low attachment U-bottom 96-well plates (174925, Nunclon TM). Scale: 10x. **(C)** Gating strategy for flow cytometry analysis for immunophenotypical characterization of MTS. The figure depicts the gating strategy for identifying BT- 474 cancer cells and CD11b^+^ myeloid cells and corresponding FMO CD206 control.


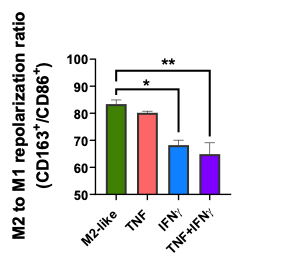


**Supplementary Figure 2. Macrophages in MTS respond to IFNγ with increased M1 polarization.** Quantification of flow cytometry M2 to M1 ratios CD163^+^CD11b^+^ (M2-like) and CD86^+^CD11b^+^ (M1-like) macrophages from 7 days growing MTS at 1 day of treatment as indicated: M2-like (untreated), TNFα (20 ng mL^-1^), IFNγ (30 ng mL^-1^) and with combination of both TNFα and IFNγ. Statistical analysis was performed by one-way ANOVA followed by Tukey’s multiple comparisons. Results are considered significant with at least p < 0.05 (*) and p < 0.01 (**). Results are expressed in duplicates as mean ± standard deviation (n=1).

**
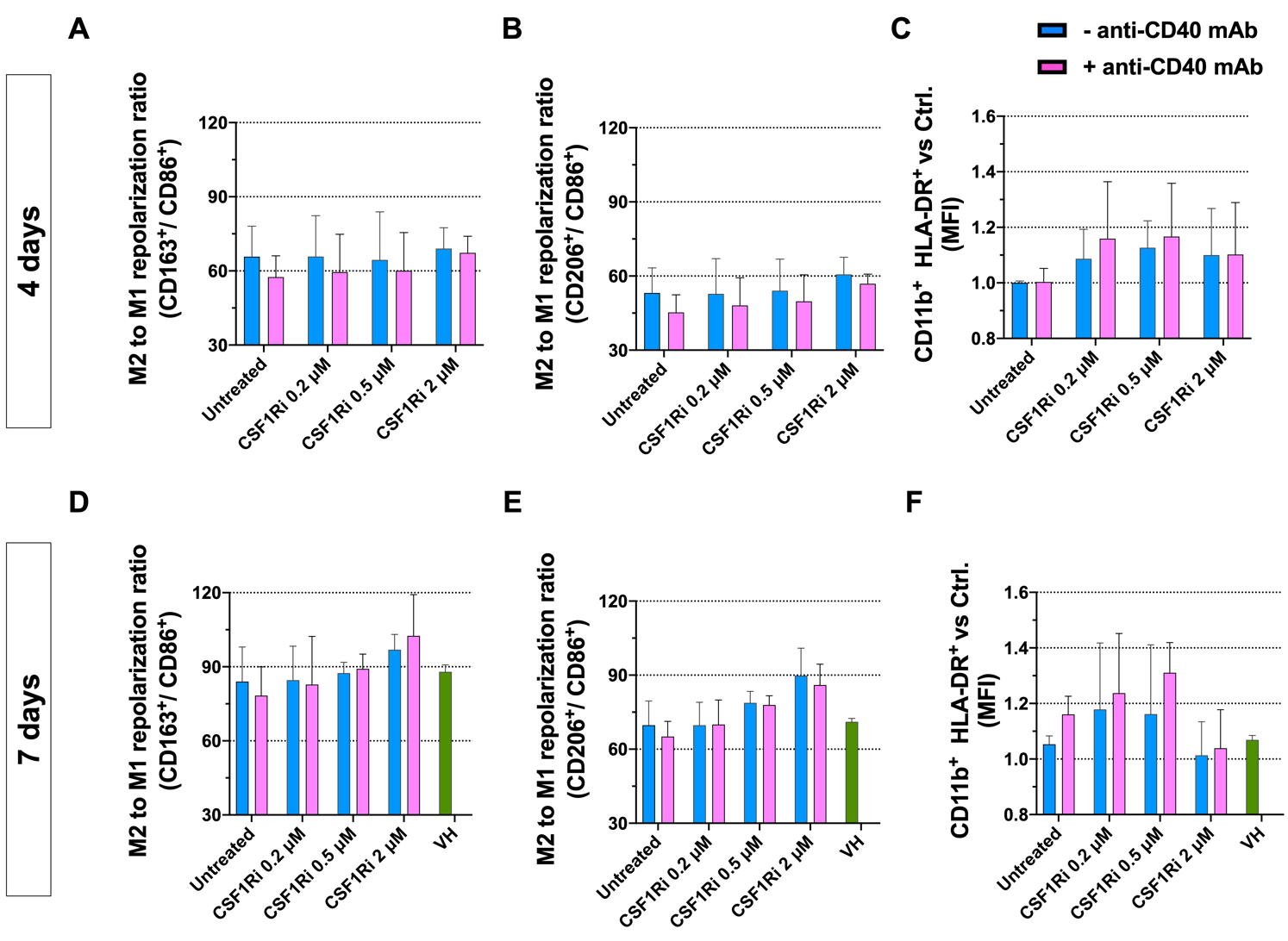
**

**Supplementary Figure 3. Flow cytometry-based analysis of M2 to M1 macrophage ratios and activation in response to anti-CD40 Ab and CSF1Ri treatments. (A, D)** CD163^+^CD11b^+^ (M2-like)/and CD86^+^ CD11b^+^ (M1-like) macrophages ratio; **(B, E)** CD206^+^CD11b^+^ (M2-like)/CD86^+^ CD11b^+^ (M1-like) macrophages ratio; **(C, F)** and HLA-DR expression (Mean Fluorescence Intensity) on CD11b^+^ macrophages from MTS, at 4 days **(A-C)** and 7 days **(D-F)** of combinatorial treatment as indicated: untreated, 0.2, 0.5 and 2 µM inhibitor of CSF1R (CSF1Ri) without (-anti-CD40 mAb) and with combination of 50 nM anti-CD40 mAb (+anti-CD40 mAb). Vehicle control (VH) treatment (1:3 mixture of THF/H_2_O v/v used to dissolve CSF1Ri). All data are presented as the mean ± standard deviation (n =3).


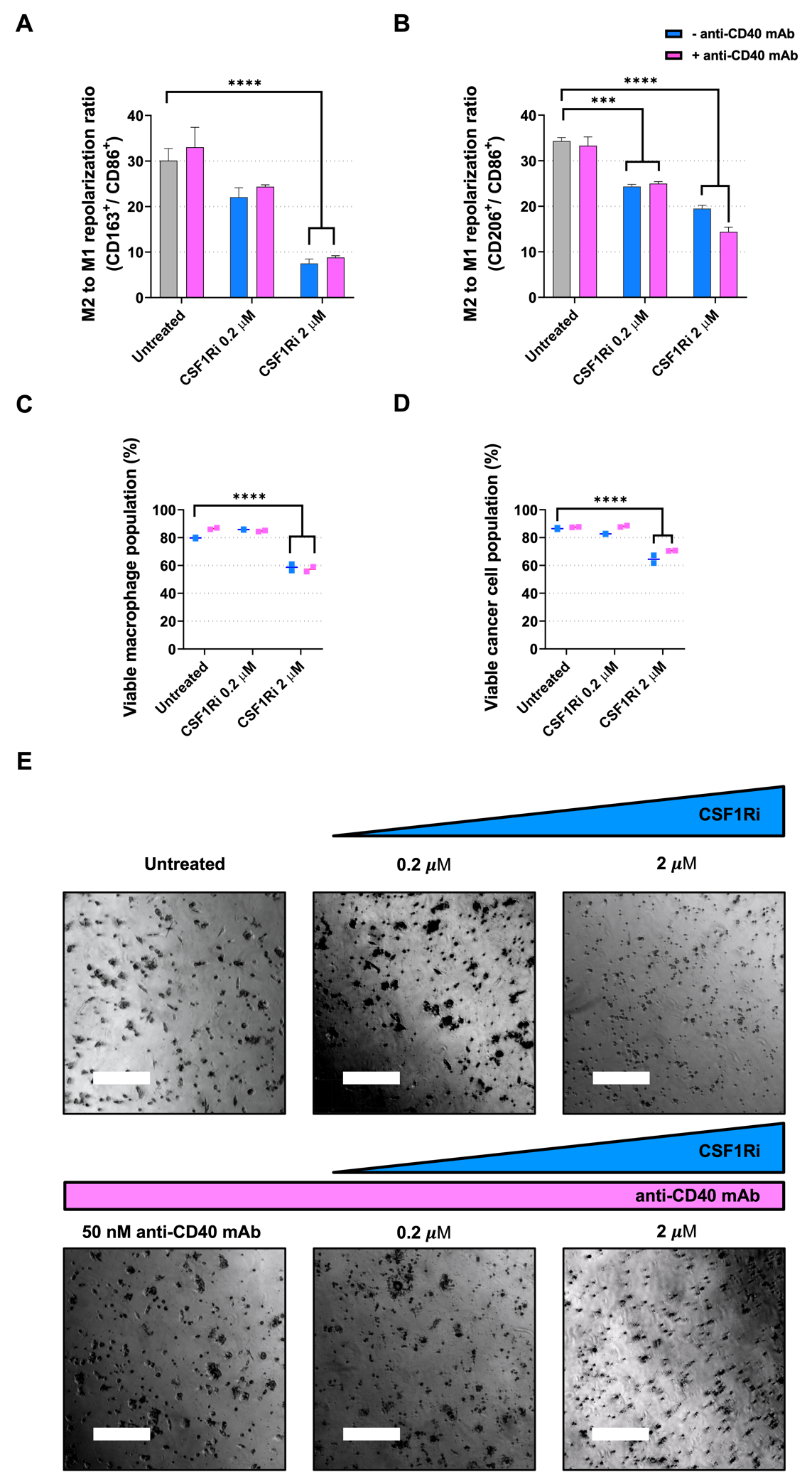


**Supplementary Figure 4. Anti-CD40 mAb and CSF1Ri treatments decrease M2-like immunophenotype and reduce macrophage and cancer cell viability in 2D macrophage-cancer cell co-culture experiments.** Flow cytometry analysis results showing ratio of **(A)** CD11b^+^CD163^+^ (M2-like) over CD11b^+^CD86^+^ (M1-like) macrophages; **(B)** CD11b^+^CD206^+^ (M2-like) over CD11b^+^CD86^+^ (M1-like) macrophages; **(C)** macrophage viability and **(D)** cancer cell viability after 3 days of agonistic anti-CD40 mAb - CSF1Ri treatment as indicated: untreated, 0.2, 2 µM of CSF1R (CSF1Ri) without (-anti-CD40 mAb) and with (+anti-CD40 mAb) combination of 50 nM anti-CD40 mAb. Results are given in percentage (viable cells) of Propidium Iodine (PI) negative vs total cancer cells; and PI negative - CD11b^+^ macrophage vs total macrophage populations. Statistical analysis was performed by two-way ANOVA followed by Tukey’s multiple comparisons. Results were considered significant with at least p < 0.001 (***) and p < 0.0001 (****) vs untreated control. Results are expressed in duplicates as mean + standard deviation (n=1). **(E)** Representative pictures of 2D macrophage- cancer cell co-cultures upon CSF1Ri and anti-CD40 mAb treatments. Conditions: untreated, 0.2, 2 µM inhibitor of CSF1R (CSF1Ri) without and with (+anti-CD40 mAb) 50 nM anti-CD40 mAb. Scale bar: 200 μm.


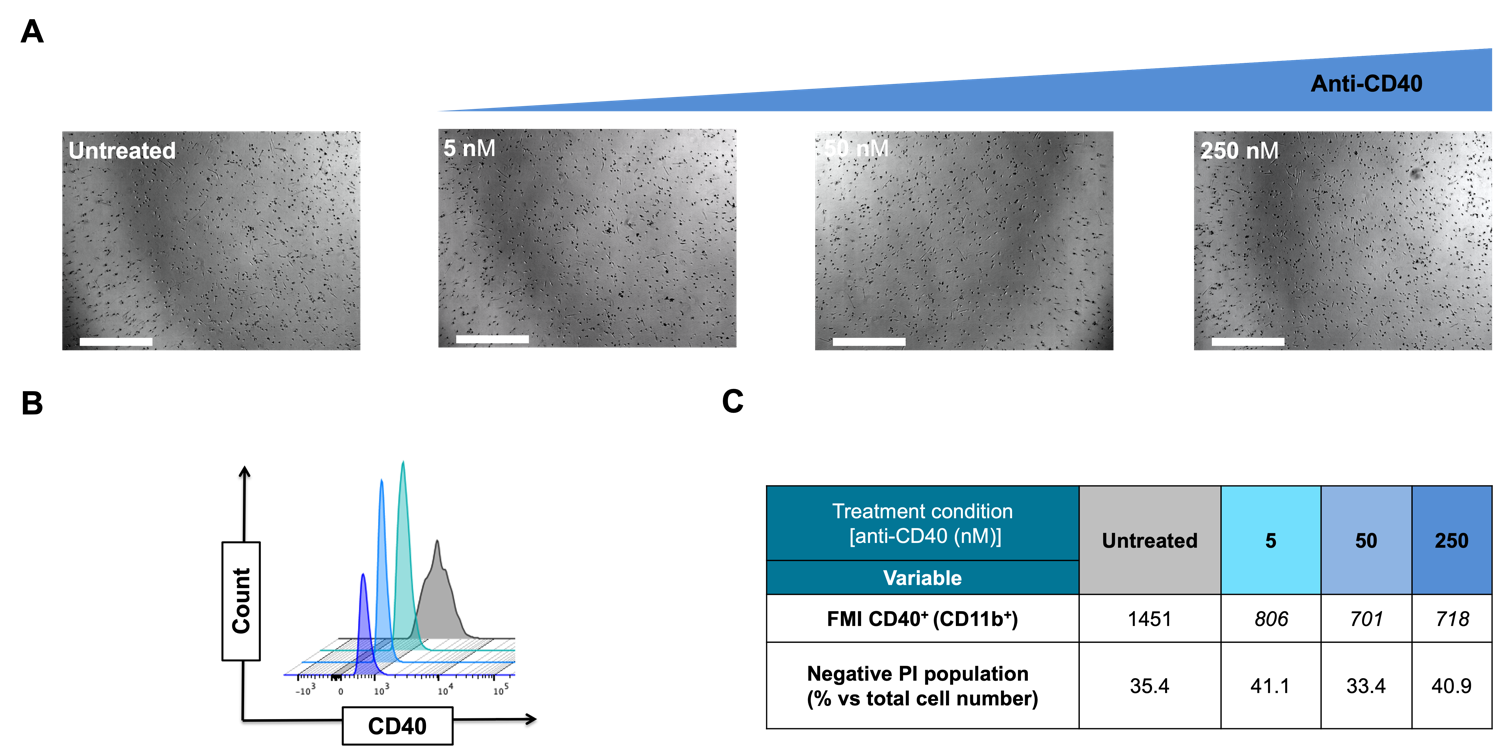


**Supplementary Figure 5. 2D monocultures of M2-like macrophages exposed to anti-CD40 mAb downregulate CD40 expression.** **(A)** Representative pictures of macrophage monocultures in 2D upon 3 days treatment with anti-CD40 mAb: untreated, 5, 50 and 250 nM anti-CD40 mAb. Scale bar: 300 μm. **(B)** Histograms of the flow cytometry analysis of CD40 expression on cultures CD11b^+^ macrophages treated as indicated. **(C)** Table showing fluorescence mean intensity (FMI) values of CD40 expression on CD11b^+^ macrophages are relative viability (% of Propidium Iodine (PI) negative stained macrophages).

**
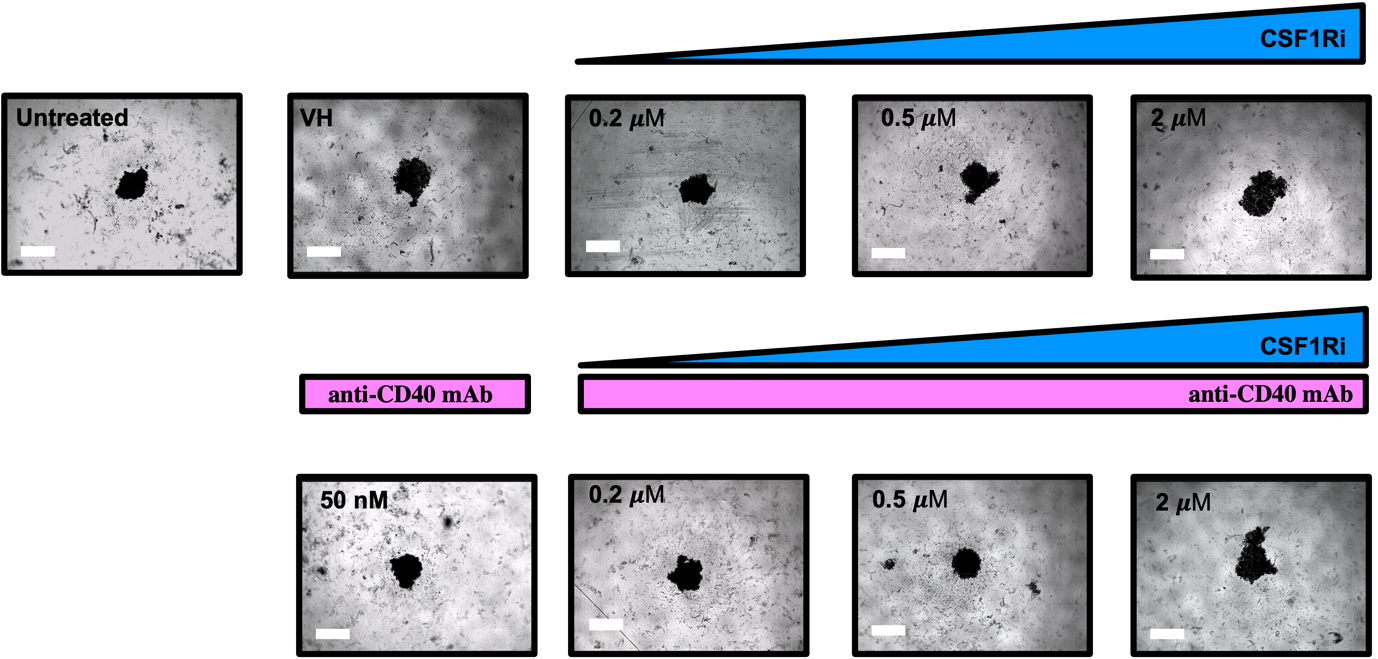
**

**Supplementary Figure 6. Representative pictures of MTS cultures after 7 days of combinatorial CSF1Ri- anti-CD40 mAb treatment:** untreated, vehicle only (VH), 0.2, 0.5 and 2 µM inhibitor of CSF1R (CSF1Ri) alone (upper row), or in combination with of 50 nM anti-CD40 monoclonal antibody (anti-CD40 mAb) (lower row). Scale bar: 300 μm.

**
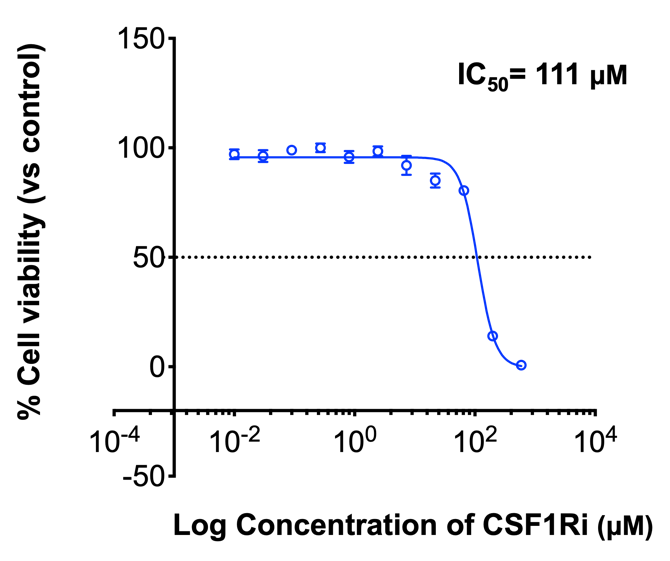
**

**Supplementary Figure 7. Determination of IC_50_ for CSF1Ri (BLZ945) treatment of the BT-474 cell line.** Each data point represents the average of quadruplicate values ± standard deviation obtained (n=2). The IC_50_ value was calculated from a log ([BLZ-945]) vs normalized response curve fit using Graphad Prism 9.0.2 for Mac.
